# Thyroseq v3, Afirma GSC, and microRNA Panels Versus Previous Molecular Tests in the Preoperative Diagnosis of Indeterminate Thyroid Nodules: A Systematic Review and Meta-Analysis

**DOI:** 10.3389/fendo.2021.649522

**Published:** 2021-05-13

**Authors:** Cristina Alina Silaghi, Vera Lozovanu, Carmen Emanuela Georgescu, Raluca Diana Georgescu, Sergiu Susman, Bogdana Adriana Năsui, Anca Dobrean, Horatiu Silaghi

**Affiliations:** ^1^ Department of Endocrinology, “Iuliu Hatieganu” University of Medicine and Pharmacy Cluj-Napoca, Cluj-Napoca, Romania; ^2^ International Institute for the Advanced Studies of Psychotherapy and Applied Mental Health, Babeş-Bolyai University, Cluj-Napoca, Romania; ^3^ Department of Morphological Sciences-Histology, “Iuliu Hatieganu” University of Medicine and Pharmacy, Cluj-Napoca, Romania; ^4^ Department of Pathology, IMOGEN Research Center, Cluj-Napoca, Romania; ^5^ Department of Community Health, “Iuliu Hatieganu” University of Medicine and Pharmacy Cluj-Napoca, Cluj-Napoca, Romania; ^6^ Department of Clinical Psychology and Psychotherapy, Babeş-Bolyai University, Cluj-Napoca, Romania; ^7^ Department of Surgery V, “Iuliu Hatieganu” University of Medicine and Pharmacy Cluj-Napoca, Cluj-Napoca, Romania

**Keywords:** thyroid cancer, TBSRTC, indeterminate cytology, diagnostic accuracy, NIFTP, molecular testing, Afirma, Thyroseq

## Abstract

**Background:**

Molecular tests are being used increasingly as an auxiliary diagnostic tool so as to avoid a diagnostic surgery approach for cytologically indeterminate thyroid nodules (ITNs). Previous test versions, Thyroseq v2 and Afirma Gene Expression Classifier (GEC), have proven shortcomings in malignancy detection performance.

**Objective:**

This study aimed to evaluate the diagnostic performance of the established Thyroseq v3, Afirma Gene Sequencing Classifier (GSC), and microRNA-based assays versus prior iterations in ITNs, in light of “rule-in” and “rule-out” concepts. It further analyzed the impact of noninvasive follicular thyroid neoplasm with papillary-like nuclear features (NIFTP) reclassification and Bethesda cytological subtypes on the performance of molecular tests.

**Methods:**

Pubmed, Scopus, and Web of Science were the databases used for the present research, a process that lasted until September 2020. A random-effects bivariate model was used to estimate the summary sensitivity, specificity, positive (PLR) and negative likelihood ratios (NLR), and area under the curve (AUC) for each panel. The conducted sensitivity analyses addressed different Bethesda categories and NIFTP thresholds.

**Results:**

A total of 40 eligible studies were included with 7,831 ITNs from 7,565 patients. Thyroseq v3 showed the best overall performance (AUC 0.95; 95% confidence interval: 0.93–0.97), followed by Afirma GSC (AUC 0.90; 0.87–0.92) and Thyroseq v2 (AUC 0.88; 0.85–0.90). In terms of “rule-out” abilities Thyroseq v3 (NLR 0.02; 95%CI: 0.0–2.69) surpassed Afirma GEC (NLR 0.18; 95%CI: 0.10–0.33). Thyroseq v2 (PLR 3.5; 95%CI: 2.2–5.5) and Thyroseq v3 (PLR 2.8; 95%CI: 1.2–6.3) achieved superior “rule-in” properties compared to Afirma GSC (PLR 1.9; 95%CI: 1.3–2.8). Evidence for Thyroseq v3 seems to have higher quality, notwithstanding the paucity of studies. Both Afirma GEC and Thyroseq v2 performance have been affected by NIFTP reclassification. ThyGenNEXT/ThyraMIR and RosettaGX show prominent preliminary results.

**Conclusion:**

The newly emerged tests, Thyroseq v3 and Afirma GSC, designed for a “rule-in” purpose, have been proved to outperform in abilities to rule out malignancy, thus surpassing previous tests no longer available, Thyroseq 2 and Afirma GEC. However, Thyroseq v2 still ranks as the best rule-in molecular test.

**Systematic Review Registration:**

http://www.crd.york.ac.uk/PROSPERO, identifier CRD42020212531.

## Introduction

Thyroid cancer (TC) accounts for 2% of all cancers and it is the most frequent endocrine malignancy. In the last decades, its incidence has increased due to improved screening and ultrasound (US) surveillance of thyroid nodules (TNs) (1, 2). Distinguishing benign from malignant disease is typically achieved by fine-needle aspiration (FNA) biopsy and cytologic evaluation of TNs based on US appearance and nodule size.

The Bethesda System for Reporting Thyroid Cytopathology (TBSRTC) argued in favor of an appreciable framework to standardize the reporting of FNA cytology results (3) and, therefore, it has become an effective tool for identifying the malignancy risks, types of neoplasms and guided clinical management. This approach reliably establishes a benign or malignant nodule diagnosis in 70 to 80% of all cases (4). However, for the remaining 20 to 30% of nodules, the FNA diagnosis falls in an interpretive gray zone, consisting of one of three indeterminate cytology categories (3, 5), i.e., follicular lesion of undetermined significance/atypia of uncertain significance (FLUS/AUS, Bethesda category III), follicular neoplasm/suspicious for follicular neoplasm (FN/SFN, Bethesda category IV), and suspicious for malignancy (SM, Bethesda category V), with a predicted probability of cancer of 10–30, 25–40, and 50–75%, respectively (3).

Historically, indeterminate thyroid nodules (ITNs) commonly underwent repeat FNA or diagnostic surgery, typically lobectomy. Approximately three-quarters of these were benign on surgical pathology, indicating unnecessary surgical removal (6). Advances in the genetics of thyroid tumorigenesis have led to the development of a series of molecular tests to complement cytology and improve the risk-based stratification of ITNs (7).

Afirma Gene Expression Classifier (GEC) from Veracyte Inc. is a microarray-based test with a proprietary algorithm that analyses the mRNA expression of a panel of 167 genes (8). Previous works report a quite high sensitivity (SE) but low specificity (SP) for Afirma GEC, making it a good “rule-out” test (9).

The ThyroSeq panel is a next-generation sequencing (NGS)-based assay that underwent several iterations over the years (10–12). ThyroSeq v2, replaced in 2011 the so-called seven-gene panel (BRAF, RAS, RET/PTC, and PAX8/PPAR) and queried 56 genes for point mutations, fusions, and abnormal gene expression. Its initial validation study claimed the potential for use as an all-around test of malignancy in ITNs given the reported positive predictive value (PPV) of 83% (13).

Recent refinements led to the development of novel analytic panels, such as Thyroseq v3 and Afirma Gene Sequencing Classifier (GSC), which became available for clinical use in 2017. Thyroseq v3 assays for a panel of 112 gene point mutations, insertions, deletions, copy number alterations, fusions, and gene expression alterations associated with TC (14, 15). The next-generation molecular tool, Afirma GSC, was released to improve the GEC’s SP and incorporated additional components for BRAFV600E mutation, RET/PTC fusion, parathyroid tissue, and medullary thyroid cancer (MTC) (16). Data from an academic center suggest an improved SP and PPV while maintaining high SE and NPV and reducing the surgery rate for GSC (17). In May 2018, Veracyte Inc. launched the Afirma Xpression Atlas (XA) which uses RNA sequencing to detect gene variants and fusions, being conceived for Afirma GSC suspicions and Bethesda V-VI lesions (18). Subsequent augmentation of the panel meant to include 905 variants and 255 fusions from 593 genes has broadened its initial use from surgical decision-making in ITNs to targeted therapies for metastatic TC (19, 20).

A multiplatform approach (MPT, Interpace Diagnostics) combines a mutation panel (ThyGenX) and a microRNA (miRNA) classifier test (ThyraMIR) that has been shown to provide both high NPV and PPV (21, 22). In the current MPT, designated MPTX, an analytically validated expanded NGS test (ThyGeNEXT), is combined with ThyraMIR. This multiplatform test demonstrated a high PPV of 75% and NPV of 97%, comparable with other marketed tests (14, 16, 23). RosettaGX Reveal (Rosetta Genomics) is a thyroid miRNAs classifier for the stratification of ITNs by evaluating the expression of 24 up and down-regulated miRNAs species, using the routinely stained cytology smears as testing substrate (24).

Currently, the AUS/FLUS category represents “the grey zone” of thyroid cytology, comprising a heterogeneous set of cases of uncertain interpretation. This feature can explain in part the more variable AUS/FLUS risk of malignancy compared to other indeterminate categories. Moreover, little is known about the impact of the molecular diagnosis on AUS/FLUS subcategorization. Recent studies have shown that the BRAFV600E mutation is more frequently associated with cytologic atypia than other qualifiers, whereas the molecular landscape of other AUS/FLUS subcategories is still evolving (25). The development of a hybrid AUS/FLUS subclassification system integrating the atypia qualifiers and molecular alteration could improve malignancy risk stratification and could also contribute to customizing the management of AUS/FLUS patients by selecting those more suitable for surgery or clinical follow-up (26). Thus, it was proposed that *BRAF*, *RAS*, *RET/PTC* alterations could be analyzed firstly if cytological atypia predominates. Conversely, if the predominant cytological features are non-typical microfollicular structures, then *RAS* and *PAX8/PPARg* alterations could be searched first (27).

Recently, a new histological category of Noninvasive Follicular Thyroid Neoplasms with Papillary-like Nuclear Features (NIFTP) was introduced to distinguish the non-invasive encapsulated follicular variant of papillary thyroid cancer (EFVPTC) from other aggressive forms of papillary thyroid carcinomas (PTC). In this original study, no adverse outcomes were found in 109 NIFTP patients, thus NIFTP was considered a lesion with an excellent prognosis appreciated currently as a low-risk thyroid neoplasm (28). Although two subsequent studies have reported a risk of lymph node and lung metastases in about 5 and 1% of the NIFTP cases, respectively (29, 30), these findings were not confirmed in the majority of cohorts after a long follow-up (31–36). Newly proposed additional diagnostic criteria for NIFTP reflect a joint effort by experts to further refine the NIFTP such that the histomorphology would correlate with an indolent outcome of this entity (37). Reliable criteria that could conduct to a diagnosis of NIFTP for cytological specimens is expected, to avoid over-treatment and additional follow-up. Also, given that some molecular tests were developed and validated before this reclassification, their performance measures have been shown to deteriorate significantly when the NIFTP designation is incorporated in the classification of ITNs (38–40).

A few previous meta-analyses have been done on this topic; most of them only analyzed single molecular testing, and none of them evaluated qualitatively the newest emerging panels, Thyroseq v3 and Afirma GSC (9, 41–43). Therefore, the present study aimed to measure the accuracy of recently developed Thyroseq v3, Afirma GSC, Interpace Multiplatform tests, and RosettaGX for diagnosis of ITNs, compare them with the initial versions and highlight each diagnostic potential in light of “rule-in” and “rule-out” concepts. The secondary aim was to perform an up-dated analysis of Thyroseq 2 and Afirma GEC and assess the impact of NIFTP reclassification, TBSRTC cytological subtypes, and industry sponsorship on the performance of these molecular tests.

## Methods

### Protocol and Registration

The protocol of the current systematic review and meta-analysis can be accessed on the Prospero website https://www.crd.york.ac.uk/prospero/ with the following registration number: CRD42020212531.

### Search Strategy

The research followed the Preferred Reporting Items for Systematic Reviews and Meta-Analyses (PRISMA) guidelines (44). We used the PICO (population, index, comparator, outcomes) system to describe the essential items for framing this review and its objective and methodology. Papers published before September 05, 2020, were searched on PUBMED, Web of Science, and Scopus databases combining the concepts “molecular panels” with “thyroid nodules” and “indeterminate cytology”. After that, we used the following search strategy on Medline: [Thyroseq OR (Afirma AND (“gene expression classifier” OR “Genomic Sequencing Classifier” OR GEC OR GSC)] OR Rosettagx OR Thyramir OR ThygenX OR (Multiplatform AND test*) OR MPTX OR ThyGeNEXT) AND [(thyroid AND (Nodule* OR tumor*)] OR indetermin* OR undetermin* OR “fine needle aspiration” OR FNAC* OR [(Bethesda OR categor*) adj6 (III OR IV OR V OR 3 OR 4 OR 5)] OR AUS/FLUS OR FN/SFN OR “suspicion of follicular neoplas*”). The search strategy in other databases was similar, following the same principles and steps. At the same time, the reference lists of review papers and original reports were hand-searched for further relevant studies. No language, publication date, or status restrictions were used.

### Inclusion Criteria

To be included in the meta-analysis, studies had to meet the following criteria:

- longitudinal studies in which individuals with nodular thyroid disease (solitary or multinodular) found by palpation or on the US, in whom FNA biopsy was performed and the categories III, IV, or V, were identified according to TBSRTC;
- studies evaluating at least one of the following molecular panels: Thyroseq, Afirma GEC or GSC, RosettaGX Reveal, ThyraMIR/ThyGenX, ThyraMIR/ThyGeNEXT (Interpace, MPTX), or miRInform;
- studies that used a histopathological examination of the thyroid surgery as the reference standard;
- studies with sufficient data [true positives (TPs), false positives (FPs), true negatives (TNs), and false negatives (FNs)] to calculate the SE, SP, positive likelihood ratio (PLR), NLR, diagnostic odds ratio (DOR), positive predictive value (PPV), negative predictive value (NPV) and benign call rate (BCR).

### Exclusion Criteria

- studies that used standard references other than histopathological examination, such as clinical or US surveillance;
- duplicates, reviews, comments, editorials, conference abstracts, and unpublished articles;
- studies that enrolled patients with benign or malignant cytology of the TNs and participants with non-diagnostic results of the molecular tests.

### Data Extraction

Two reviewers (SCA, LV), working independently, read the included articles’ titles and abstracts and judged their eligibility. A third investigator (SH) adjudicated any discrepancies. After excluding papers that did not meet our inclusion criteria, we read the full texts, and relevant data were extracted and tabulated in a Microsoft Excel sheet framework. The following items were eligible to collect and record for each manuscript:

- publication information (first author, publication year, country of origin);
- patients’ characteristics (participants’ and TNs number, mean age, gender ratio);
- index test information (the molecular panel);
- reference standard information (histological subtypes after surgical treatment, number of NIFTP cases and their index test results);
- study flow and timing (number of FNA biopsies performed to confirm indeterminate cytology, percentage of resected nodules among the entire cohort, group with the positive and negative index test result, number of nodules with non-diagnostic test result);
- statistical analysis (TPs, FPs, TNs, and FNs).

When the appropriate size effect was not available, original data had been extracted from the article to calculate them, or we contacted the authors to offer the missing data.

### Assessment of Methodological Quality

Two reviewers (SCA, LV) assessed the studies’ quality using the Quality Assessment of Diagnostic Accuracy Studies 2 (QUADAS-2) (45). The domains included in the risk of bias and applicability evaluation were participant recruitment, index test, reference standard, flow, and timing. We customized the signaling questions for each of the four QUADAS-2 domains (**Supplementary Table 1**). According to the signaling questions, the risk of bias and applicability were evaluated as low, high, or unclear (**Supplementary Table 2**). For each signaling question, reviewers were required to answer “yes,” “no,” or “unclear.” Divergent answers among reviewers were resolved through discussions. No study was excluded as a result of findings from the risk of bias assessments. However, due to the limited number of studies labeled with a low risk of bias, we could not synthesize separately the results for this subgroup.

### Statistical Methods

For each panel, the TP, FP, TN, and FN were used for computing SE, SP, PLR, NLR, and DOR. SE and SP with their corresponding 95% confidence intervals (CIs) were used to pooled data using the bivariate random-effects model. The analyses were done using MIDAS from STATA software (version 16.0), which uses joint modeling SE and SP. The pooled PLR was derived to describe the ratio of a positive outcome in cancer cases, while the pooled NLR the ratio of a positive outcome in those without cancer. DOR, the odds of PLR to NLR, ranging from zero to infinity, were derived to estimate the diagnostic accuracy. Also, PPV as the proportion of individuals with positive test results who are correctly identified as having malignant disease and NPV as the proportion of patients with negative test results who truly have benign nodules were calculated. When we have computed the PPV and NPV estimates we quantified the prevalence in a given population by specifying a prior distribution, f (p), on p, following the recommendations described by Li et al. (46). Specifically, we have estimated the prevalence in each study and used the lowest/highest prevalence rates as interval limits in pddam command from midas (i.e., midas tp fp fn tn, pddam (lbp ubp). Finally, we determined the benign call rate (BCR) as the percentage of molecular tests that result in a benign or negative test result.

For providing inferences regarding diagnostic quality, we plotted a Summary Receiver Operating Characteristic (SROC) curve for each panel. The area under the curve (AUC) was used to estimate the panel’s diagnostic accuracy. Furthermore, we had conducted a series of sensitivity analyses looking at the pooled SE and SP when NIFTP was excluded from the malignant histologies, at different Bethesda categories, at studies in which the authors were paid as employees of a pharmaceutical company.

We assessed heterogeneity across studies through the I² statistic, and we used a bagplot to examine the spread of the observed data and identify outliers. We examined each panel’s clinical utility using Fagan plots with pre-specified probabilities of 25, 50, and 75% respectively. Evidence of publication bias was assessed through Deeks’s funnel plot.

### Ethical Approval

This article does not contain examinations performed on human participants. Thus, ethical approval was not necessary.

## Results

### Literature Search

**Figure 1:**
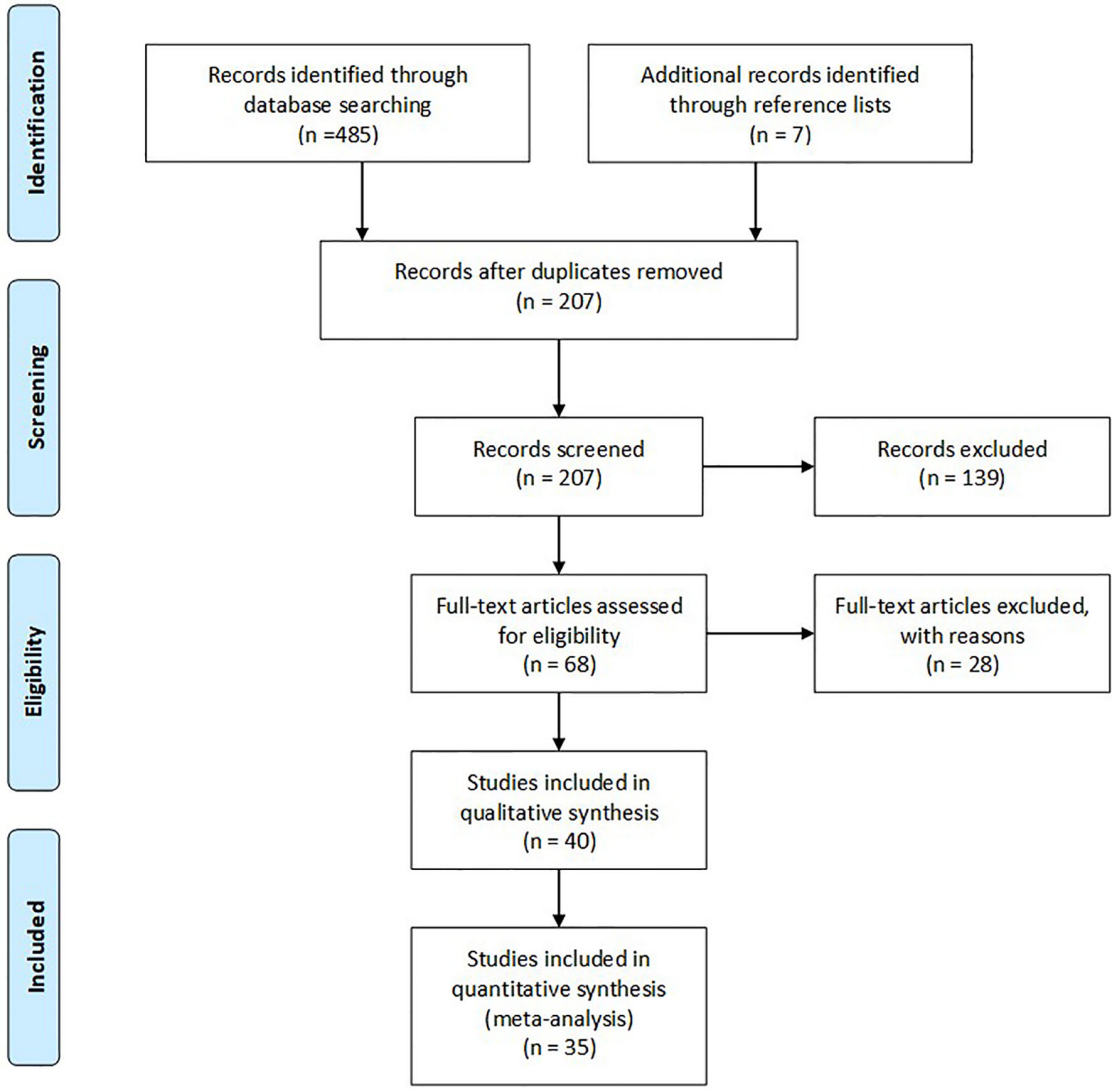
PRISMA flowchart of the included studies.

Our literature search in PUBMED, Web of Science, and Scopus databases until September 05, 2020, identified 485 potentially relevant publications. An additional seven studies were found, besides by hand-searched of the review papers and original reports. After removing duplicates, we identified 207 abstracts. We excluded a total of 139 records as they represented irrelevant studies to the current analysis, papers with clinical and US follow-up only as of the reference standard, evaluation of different preparation smears, studies evaluating lymph nodes or residual FNA rinse samples, analytical validation studies, review articles, case reports, comments, letters or reply. The remaining 68 pieces were deemed relevant by title and abstract alone. Based on the readings of the full-text articles, we excluded 28 articles for reasons. **Figure 1** illustrates the Preferred Reporting Items for Systematic Reviews and Meta-Analyses (PRISMA) flow-chart of the study selection process.

### Participant and Study Characteristics

We included in the review a total of 40 articles from the USA with 50 assessments of association between seven molecular panels and postsurgical histological evaluation (8, 11, 14–17, 21, 23, 40, 47–77). **Table 1** summarizes the characteristics of the included studies. All 40 articles are published in English. The publication year ranged from 2012 to 2019, while the populations were enrolled between September 2009 to June 2019. All but one study were conducted in the USA, with the originates in Singapore (73). A minority of the studies had a prospective design (n = 10) (8, 14, 15, 21, 50, 55, 57, 64, 77), of which one research performed a parallel randomized study (61), and another two studies enrolled patients both retro- and prospectively (11, 75).

**Table 1:** Characteristics of the included studies.

Author, year	Panel	Participants no.	Female (%)	Mean age	Nodule size (cm)	Bethesda category	NIFTP no. (result)	Conflicts of interest
**Alexander, 2012** (8)	AGEC	379	81.7	53,2	2,2	III, IV, V	N/A	Yes
**Alexander, 2014** (47)	AGEC	339	79	55	2,2	III, IV, V	N/A	Yes
**Al-qurayshi, 2016** (48)	AGEC	145	73	56,0	2,4	III, IV	N/A	Yes
**Arosemena, 2019** (49)	AGEC	117	80	58,0	2,03	III	0	No
**Azizi, 2018** (50)	AGEC	151	89	51,4	1,65	III, IV	N/A	No
**Baca, 2017** (51)	AGEC	229	78	56,3	1,8	III	15 (+)	Yes
**Brauner, 2015** (52)	AGEC	72	81	59,2	2,2	III, V (HC)	N/A	Yes
**Celik, 2015** (53)	AGEC	46	71.2	59,4	?	III, IV, V	N/A	No
**Chaudhary, 2016** (54)	AGEC	158	?	?	?	III, IV	N/A	No
**Chen, 2019** (55)	TQ3	51	74	54	2,5	III, IV	N/A	No
**Deaver, 2018** (56)	AGEC	167	?	?	?	III, IV	2 (+)	Yes
**Endo, 2019** (17)	AGEC	317	77.5	52,1	2,23	III, IV	6 (+)	No
	AGSC	153	75	54,9	2,3	III, IV	1 (+)	No
**Harrell, 2018** (57)	AGEC	481	?	?	?	III, IV	0	Yes
	AGSC	139	?	?	?	III, IV	1 (−)	Yes
**Jug, 2018** (58)	AGEC	207	78	57	1.1-5.3	III, IV, V	4 (+)	No
	TQ2	97	80	59,	1.1-5.3	III, IV, V	4 (+)	No
**Jug, 2020** (59)	TQ3	91	81	58,9	?	III, IV	2 (+)	No
	TQ2	94	80	58,1	?	III, IV	2 (+)	No
**Labourier, 2015** (21)	MPT	109	74	56	–	III, IV	N/A	Yes
**Lithwick, 2016** (60)	Rosetta	201	80	53	> 0,5	III, IV, V	N/A	Yes
**Livhits, 2018** (61)	AGEC	70	81.4	61	2,0	III, IV (HC)	6 (+)	No
	TQ2	79	83	57	2,1	III, IV	2 (+)	No
**Lupo, 2020** (23)	MPTX	197	73	55	2,3	III, IV, V	5 (+)	Yes
**Marcadis, 2018** (62)	TQ2	266	75	53	2,7	III, IV	38 (+), 8 (−)	No
**Marti, 2015** (63)	AGEC	156	88.4	51.7	2.17	III, IV	N/A	No
**Mciver, 2014** (64)	AGEC	72	?	?	?	III, IV (HC)	N/A	No
**Nikiforov, 2014** (11)	TQ2	143	?	?	?	IV	N/A	Yes
**Nikiforov, 2015** (15)	TQ2	441	?	?	?	III	N/A	Yes
**Nikiforova, 2018** (65)	TQ3	175	?	?	?	III, IV, V	6 ()?	Yes
**Papoian, 2020** (66)	AGEC	69	87	50	1,9	III	N/A	No
**Partyka, 2019** (68)	AGEC	68	75	12-81	?	III, IV	4 (+)	No
	MPT	22	63	18-77	?	III, IV, V	1 (+)	No
	Rosetta	23	78	19-68	?	III, IV, V	1 (+)	No
**Partyka, 2018** (67)	AGEC, MPT, Rosetta	10	70	25-65	?	III, IV, V	N/A	No
**Patel, 2018** (16)	AGSC	183	77.6	51,7	2,6	III, IV	N/A	Yes
**Sacks, 2016** (69)	AGEC	140	73,5	58,	2	III, IV	N/A	Yes
**Samulski, 2016** (40)	AGEC	294	?	?	?	III, IV, HC	10 (+)	Yes
**San Martin, 2019** (70)	AGEC	178	63	59	2,0	III, IV	N/A	No
	AGSC	121	75	56,1	2,0	III, IV	N/A	No
**Shrestha, 2016** (71)	TQ2	45	77,2	48	?	III, IV, V	N/A	Yes
**Steward, 2018** (14)	TQ3	232	79	53,0	2,4	III, IV, V	11 (+)	Yes
**Sultan, 2016** (72)	AGEC	48	80	54,3	2,49	III, IV	4 (+)	No
**Taye, 2018** (73)	TQ2	151	79	52	2,6	III, IV	2 (+), 1(−)	No
**Valderrabano, 2016** (74)	miRInform	105	82	56	2,6	III, IV, V	N/A	Yes
**Valderrabano, 2017** (75)	TQ2	182	76	56,2	?	III, IV	6 (+), 2(−)	Yes
**Wang, 2020** (76)	AGEC	281	81	51	2,4	III, IV	N/A	No
**Wu, 2016** (77)	AGEC	230	80	51,9	?	III, IV	N/A	Yes

AGEC, Afirma Gene Expression Classifier; AGSC, Afirma Gene Sequencing Classifier; cPTC, classic papillary thyroid cancer; E-FVPTC, Encapsulated FVPTC; FVPTC, follicular variant of papillary thyroid cancer; FTC, follicular thyroid cancer; HC, Hurthle cell predominant; HCC, Hurthle cell carcinoma; I-FVPTC, Infiltrative FVPTC; MI, minim invasive; OVPTC, Oncocytic variant papillary thyroid cancer; MPT, Multiplatform Test (ThyraMIR/ThyGenX); MPTX, ThyraMIR/ThyGeNEXT; MTC, medullary thyroid cancer; N/A, Not available; NIFTP, Noninvasive follicular thyroid neoplasm with papillary like nuclear features; no., number; PDTC, poorly differentiated thyroid cancer; Pro, Prospective, PTC, Papillary thyroid cancer; Retro, Retrospective; TC, thyroid cancer; TQ2, ThyroseQ version 2; TQ3, ThyroseQ version 3.

The analysis included a total of 7,831 TNs from 7,565 patients. The average participants’ age of the 30 articles that reported the mean or median values is 54.5 and ranges between 12 and 81 in the rest of the studies. The authors provided information on the gender of the included participants in 33 studies; the average female percentage is 79.4%. The mean diameter of the TNs among the studies that reported this parameter was 2.32 cm. Among the articles that reported the number of TNs by TBSRTC, 4,501 (67.7%), 1,911 (28.7%), and 235 (3.5%) nodules were categories III, IV, and V, respectively. In nine papers, the patients were included after the second FNA that confirmed indeterminate cytology (50–52, 54, 58, 59, 69, 70, 72). Of the 7,831 TNs included, 240 (3.1%) have been proven to be non-diagnostic according to the molecular test result; therefore, we excluded them from the final analysis.

Regarding molecular panels, 25 studies evaluated the diagnostic performance of the Afirma GEC (8, 16, 17, 40, 47–54, 56–58, 61, 63, 64, 66–70, 72, 76, 77), and four articles reported the Afirma GSC (16, 17, 57, 70). Thyroseq versions 2 and 3 were found in nine (11, 15, 58, 59, 61, 62, 71, 73, 75) and four papers (14, 55, 59, 65), respectively, while ThyraMIR/ThyGenX (23, 67, 68) and ResettaGX Reveal (61, 67, 68) were explored in three studies, respectively. The panels with the fewest assessments were ThyraMIR/ThyGeNEXT and miRInform (23, 74). The majority of the studies (n = 32) evaluate a single molecular panel, of which 18 articles reported Afirma GEC alone (8, 40, 47–54, 56, 63, 64, 66, 69, 72, 76, 77), nine papers assessed second or third version of Thyroseq NGS (11, 14, 15, 55, 62, 65, 71, 73, 75) and lastly, ThyraMIR/ThyGeNEXT, ThyraMIR/ThyGenX, miRInform and RosettaGX Reveal were each approached in a study (21, 23, 60, 74). Among the nine studies that measured up more that two molecular panels, four paper compared Afirma GEC and GSC (16, 17, 57, 70), two manuscripts reported a comparison between Afirma GEC and Thyroseq v2 (58, 61), two papers investigated the diagnostic performance of Afirma GEC, RosettaGX and Interpace MPT (67, 68) and Jug et al. (59) compared the two last versions of Thyroseq NGS. Only two studies applied different molecular tests on the same cohort (16, 67).

Among the total TNs, 4,427 (56.5%) had undergone surgical resection. Overall, the surgery rate was significantly higher when the test result was positive or suspicious (64.3%), comparing to the surgery rate in patients with negative test results (34.6%). Among the resected nodules, 1,667 (36.4%) were found malignant at the histopathological evaluation. The most frequent malignancy reported were classic PTC, follicular thyroid cancer (FTC), FVPTC, and Hurthle cell carcinoma (HCC) diagnosed in 611, 255, 95, and 53 nodules, respectively. Conversely, we found MTC and poorly differentiated thyroid cancer (PDTC) in seven cases each. Sixteen studies revealed 144 NIFTP cases after histological evaluation (14, 17, 23, 40, 49, 51, 56–59, 61, 62, 65, 67, 72, 73, 75).

From the included papers, 21 studies reported conflicts of interest, such as grant supports, sponsorship from the commercial company, the authors’ involvement as consultants or investigators at the trading laboratory, ownerships, or intellectual property related to one of the panels (8, 11, 14–16, 21, 23, 40, 47, 48, 51, 52, 56, 57, 60, 65, 69, 71, 74, 75, 77).

### Excluded Studies

Based on the readings of the full-text articles, we excluded 28 articles for the following reasons: only enrolled nodules with benign test results (n = 4) (78–81) or suspicious test results (n = 1) (82), evaluated nodules with benign or malignant cytology (n = 2) (83, 84), did not perform surgery and consequently did not provide reference standard in nodules with benign test results (n = 7) (85–91), an overlap of the participants with other studies (n = 8) (92–99), used freshly collected FNA samples as the reference standard (n = 1) (100), unavailable statistical analysis (n = 4) (13, 22, 101, 102), and unavailable full-text article (n = 1). Finally, 40 articles met initial eligibility criteria and were systematically reviewed and abstracted. We included all of them in the quantitative analysis.

### Key Results Regarding the Diagnostic Performance of the Molecular Panels

#### Afirma GEC and GSC

A total of 25 studies involving 4,538 cytologically ITNs of the 4,424 participants evaluated the Afirma GEC performance (8, 17, 40, 47–54, 56–58, 61, 63, 64, 66–70, 72, 76, 77). The recruitment period ranged from May 2009 until June 2018. The reported number of non-diagnostic results of the GEC was 181 (4.0%), ranging from 0 to 13.5% among individual studies. Slightly more nodules had a suspicious test result (55.7%) rather than negative (44.3%). The nodules’ surgery rate with valid GEC result was 57.9%, with a significant gap between resections of those with suspicious (85.0%) and negative test results (22.9%). Following surgery and histological evaluation, 895 of 2,365 nodules were malignant, with a malignancy rate among resected nodules of 37.8%. Ten studies performed histological evaluation to highlight the number of NIFTP lesions (17, 40, 49, 51, 56, 58, 61, 68, 72, 80). Thus, 54 NIFTP cases were established, all with a suspicious Afirma GEC result. GEC’s SE and SP among studies ranged from 78.0 to 100% and 7.7 to 51.7%, respectively.

Among the 25 papers that approached Afirma GEC, four studies enrolled an additional number of 635 TNs from 596 patients to evaluate the Afirma GSC (16, 17, 57, 70). The recruitment period was held from June 2009 until December 2018. The reported number of non-diagnostic results of the GSC was slightly lower (20, 3.1%), reaching the highest percentage in Patel et al. (16) (9.0%). The number of GSC negative results increased extensively to 72% comparing with GEC. The surgery rate among the nodules with valid GEC results was lower than GEC (53.3%). We have noticed a significant gap between the percentage of resected nodules with suspicious (79.7%) and negative test results (36.3%). Following surgery and histological evaluation, 125 of 310 (40.3%) nodules were found malignant and two TNs were labeled as NIFTP. GEC’s SE and SP across studies ranged from 90.6% to 100 and 28.6%to 68.3%, respectively.

#### Thyroseq Next Generation Sequencing (NGS)

Nine studies involving 1,549 Bethesda III, IV, and V TNs of 1,498 participants evaluated Thyroseq v2 (11, 15, 58, 59, 61, 62, 71, 73, 75). The recruitment period ranged from June 2012 until June 2017. The reported quality failure proportion of the Thyroseq v2 was exceptionally low (13, 0.8%). We have found negative test results in three quarters (74%) of the investigated nodules. The percentage of surgical resections among the nodules with valid Thyroseq results was 53%, with a significant gap between resections of those with high-risk test results (91%) and negative test results (39%). Following surgery and histological evaluation, 238 of 808 (29.4%) nodules were found malignant. Three studies reported the number of NIFTP lesions (14, 59, 65), of which 13 had a positive test result and for six the result was not reported. The SE and SP of Thyroseq v2 ranged from 70 to 100% and 44 to 93%, respectively, across studies.

Additional four studies, including 603 TNs from 549 patients, to evaluate the Thyroseq v3 (14, 55, 59, 65). The reported number of non-diagnostic results of the Thyroseq v3 was 33 (5.5%), ranging from 1.1 to 10.1% among individual studies. The number of Thyroseq v3 negative and positive results were approximately equal (54% *vs*. 46%). Among the nodules with valid test results, the surgery was performed in 84%, is at a high degree in both groups of patients with high (98%) and low risk (84%) test results. Following surgery, 202 of 480 nodules (42%) were found malignant and 65 cases of NIFTP were revealed. Of the patients with NIFTP histology, 54 (83%) had a positive test result. Among the studies that assessed the performance of Thyroseq v3, the SE ranged from 93.4 to 100%, while the SP varied between 16.7 and 100%.

#### Interpace Multiplatform Tests (MPTX, MPT)

The authors evaluated ThyraMIR/ThyGenX (MPT) in three studies involving 141 cytologically ITNs (21, 67, 68). The recruitment period ranged from 2011 to 2018. Interpace’s algorithm used a two-step process, through which ThyGenX is performed first. If this oncogene panel was negative or only RAS mutations were identified, a reflex ThyraMIR test would be then performed. None of the studies reported any non-diagnostic test results. The number of MPT negative and positive results were approximately equal (53% *vs*. 47%). Among the nodules with a valid test result, 95.0% underwent surgical resection. After surgical treatment, histological diagnostic revealed 43 of 135 (31.8%) malignancies. A single NIFTP case with a positive MPT test result was revealed following histological assessment (68). The SE and SP of MPT across studies ranged from 88.6 to 100% and 54.5 to 85.1%, respectively.

The most recent version of the Interpace platform ThyraMIR/ThyGeNEXT (MPTX) was approached by Lupo et al., on 197 patients enrolled from 2013 to 2019 (23). In a similar two-step approach, MPTX is reported as negative when no mutations (ThyGeNEXT) are detected and the miRNA (ThyraMIR) test is negative; as positive when a strong driver mutation is detected or when the miRNA test is positive; and as moderate when a weak driver mutation is detected and the miRNA test is negative or moderate, or when no mutations are detected and the miRNA test is moderate. All included ITNs underwent surgical resection, which revealed 115 (58.3%) malignancies and 5 NIFTP cases, all with positive MPTX diagnosis. The calculated SE and SP of MPTX are 94.3% and 61.4%, respectively.

#### MiRNA-Based Platforms: RosettaGX Reveal and miRInform

Three studies enrolled 234 cytologically ITNs and tested them with RosettaGX Reveal molecular panel from 2015 to 2018 (60, 67, 68). The reported number of non-diagnostic results was 12 (5.1%), ranging from 0 to 6.0% among individual studies. The number of RosettaGX Reveal negative and positive results were approximately equal (53% *vs*. 47%). The nodules’ surgery rate with a valid test result was 99%. After surgical treatment, the histological assessment revealed 72 of 120 (60%) malignant tumors. A single NIFTP case with a positive RosettaGX result was recorded following histological assessment (68). The SE and SP of the RosettaGX Reveal panel ranged from 85.2 to 100% and 69.2 to 85.7%, respectively, across studies.

Valderrabano et al., tested miRInform, an initial iteration of ThyraMIR, on a total of 105 Bethesda III–V nodules recruited from 2012 to 2014 (74). The surgery rate among TNs with a valid test result was 54.3%. After surgical treatment, histological appraisal revealed 26 of 63 (41.2%) neoplastic tumors. Valderabano et al. provided a 50.0% SE and an SP of 91.9%, respectively for miRInform.

### Quality Assessment

**Figure 2:**
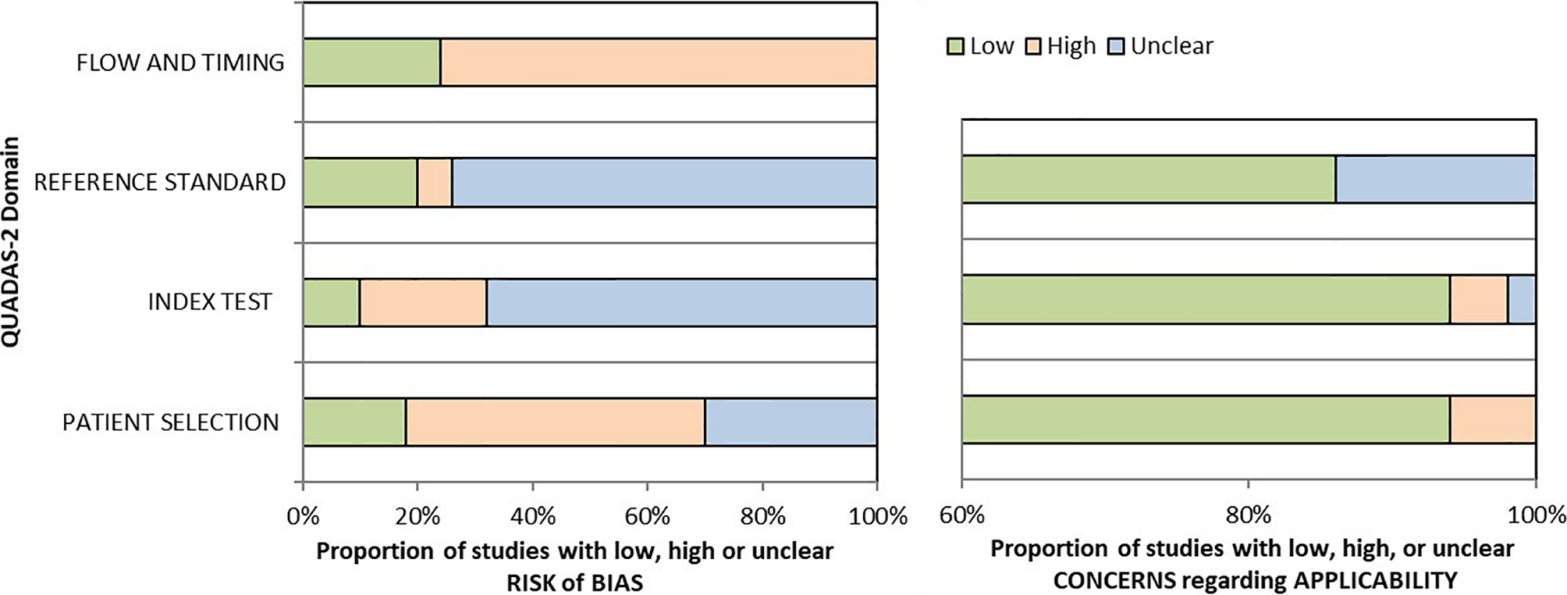
Graphical summary for risk of bias and applicability concerns using the QUADAS-2 tool.

Two reviewers (SCA and LV) critically assessed the 40 studies’ quality in the qualitative analysis using the QUADAS-2 tool (45). We used graphs (**Figure 2**) and a table (**Supplementary Table 3**) to present results for each domain’s risk of bias and applicability concerns. Since many studies evaluated multiple index tests, we divided them into several groups, one per index test, raising the total number of appraisals to 50.

We determined a high risk of bias for the “Patient selection” domain in the pooled studies due to lack of consecutive or random enrollment or inappropriate exclusion in several articles (8, 11, 14, 16, 17, 21, 49–52, 60, 62–65, 67–69, 71, 73, 76, 77). Almost all records scored an unclear risk of bias for the “Index test” domain as they did not report whether the molecular panel was interpreted without knowledge of the histopathological diagnosis (17, 20, 30, 32, 35, 38, 41–44, 46–50, 53–57, 59–61, 103). The overall risk of bias concerning the reference standard was labeled as unclear because most of the studies but nine (13, 14, 29–31, 35, 45, 49, 104) have poorly described whether the evaluators were blind to the index test results. The risk of bias for studies flow and timing was set as high as in just 13 of 50 assessments reference standard was available in all the enrolled patients (11, 14–16, 21, 23, 60, 62, 65, 67, 68, 71).

If considering the risk of bias for each molecular test, studies evaluating miRNA-based platforms and Thyroseq v3 seem to outperform in terms of flow and timing, as the histological evaluation was available for the majority of included participants. However, this relative superiority is countered by the limited number of studies for these assays. Also, miRNA-based panel Interpace has shown the lowest risk of bias concerning index test, as in two of three studies the molecular testing was performed blind to histological diagnosis (23, 67). For other criteria, the quality concerns were similarly high for all tests.

There is a low concern regarding applicability that the included patients do not match the review question as just a few manuscripts restricted the cohort to ITNs with Hurthle cell pattern (52) or Hashimoto thyroiditis (66). Besides, there is a low applicability concern that the conduct or interpretation of the index test differ from the review question in all but three articles in which the choice to order GEC or referral for surgical evaluation was made by the individual clinical provider (56) or molecular test results reported together, such as Afirma GSC with GEC (68) or Thyroseq v1 with Thyroseq v2 (71). Additionally, there is an unclear applicability concern in several studies that did not report the histological subtypes after surgical treatment (57, 58, 66, 71).

Due to the limited number of studies labeled with a low risk of bias, we could not perform sensitivity analyses to explore the influence of the studies’ quality on the results.

### Quantitative Analysis of the Molecular Panels’ Diagnostic Performance

#### Diagnostic Performance of Thyroseq v3

**Figure 3:**
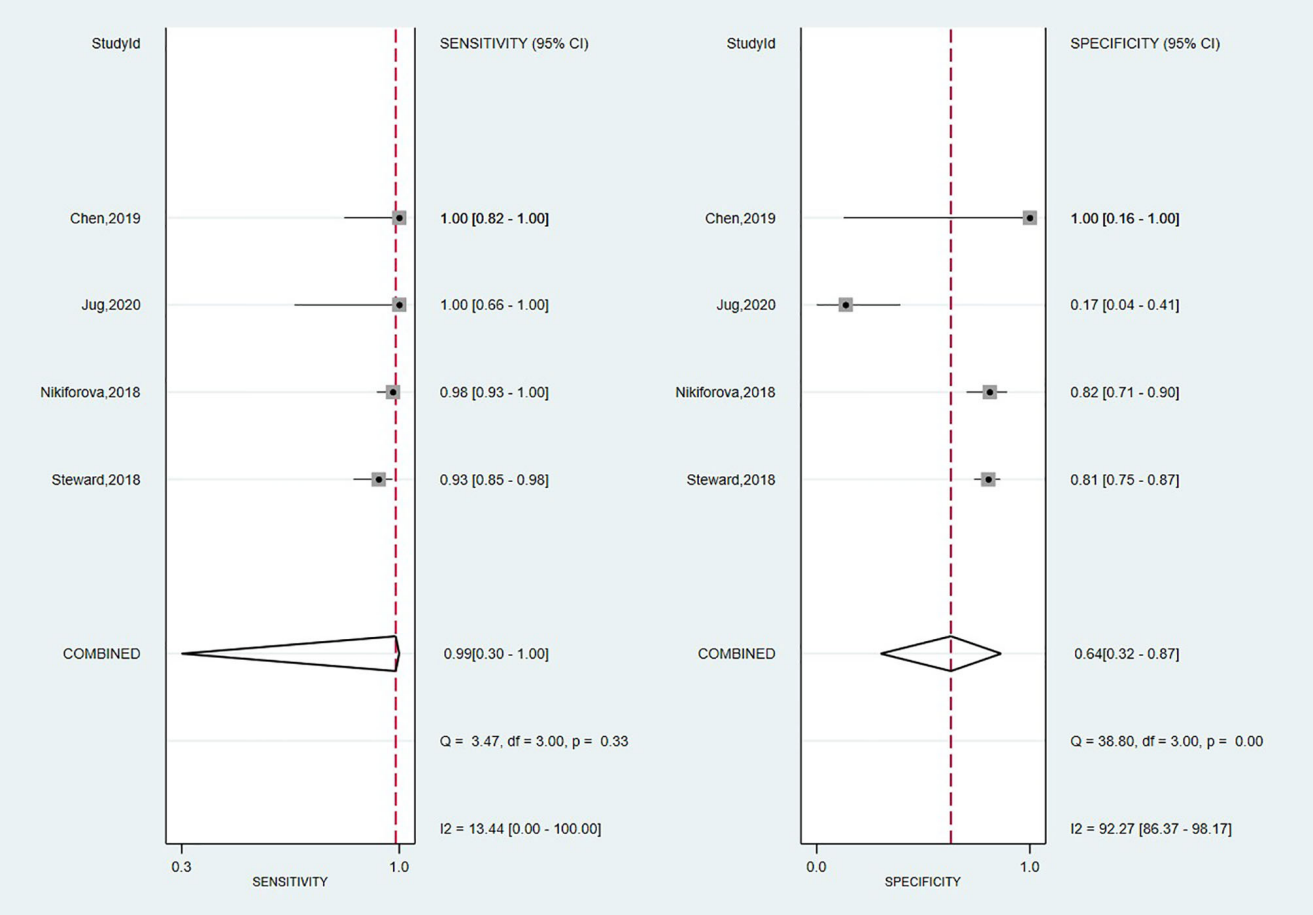
Forest plot of sensitivity and specificity for Thyroseq v3.

A total of four studies have investigated the accuracy of Thyroseq v3 in detecting malignancy (14, 55, 59, 65). The overall forest plot is shown in **Figure 3** with a SE of 0.99 (95% CI: 0.30 to 1.00), SP of 0.64 (95%, CI: 0.32 to 0.87), and a heterogeneity of I^2^ = 58%, 95% CI: 39 to 77 for SE, respectively I^2^ = 84%, 95% CI: 79 to 90 for SP. The pooled results for Thyroseq v3 shows a PPV of 0.78 (95% CI: 0.68–0.88), and NPV of 0.96 [0.83–0.88]. Thyroseq v3 indicates a PLR of 2.8 (95% CI: 1.2–6.3) and NLR of 0.02 [0.00–2.69], as displayed in **Table 2**. Additionally, we revealed a high DOR with a large 95% CI [157 (1–18,723)]. The area under the SROC curve was 0.95 (95% CI: 0.93–0.97; **Supplementary Figure 1**). The overall ThyroSeq v3 molecular test BCR was 53%. We have been unable to analyze the impact of NIFTPs, the Bethesda classification of ITNs, and declared conflicts of interest on the results for Thyroseq v3 due to a limited number of studies.

**Table 2:** Synthesis of the molecular tests’ diagnostic performance.

Panel	Thyroseq v3	Thyroseq v2	Afirma GSC	Afirma GEC
**No of studies**	4	9	4	25
**SENS [95% CI]**	0.99 [0.90–1.00]	0.86 [0.81–0.90]	0.95 [0.86–0.90]	0.97 [0.93–0.98]
**SPEC [95% CI]**	0.64 [0.32–0.87]	0.75 [0.63–0.90]	0.51 [0.33–0.69]	0.19 [0.15–0.24]
**PLR [95% CI]**	2.8 [1.2–6.3]	3,5 [2.2–5.6]	1,9 [1.3–2.8]	1.2 [1.1–1.3]
**NLR [95% CI]**	0.02 [0.00–2.69]	0.18 [0.12–0.27]	0.11 [0.04–0.27]	0.18 [0.10–0.33]
**PPV [95% CI]**	0.78 [0.68–0.88]	0.51 [0.41–0.60]	0.60 [0.52–0.68]	0.39 [0.37–0.40]
**NPV [95% CI]**	0.96 [0.83–0.88]	0.95 [0.85–1.00]	0.91 [0.80–0.68]	0.91 [0.88–0.93]
**DOR [95% CI]**	157 [1–18723]	19 [9–42]	18 [6–53]	7 [3–13]
**AUC [95% CI]**	0.95 [0.93–0.97]	0.88 [0.85–0.90]	0.90 [0.87–0.92]	0.60 [0.56–0.65]
**BCR**	0.53	0.74	0.73	0.42

AUC, area under the curve; CI, confidence interval; DOR, diagnostic odds ratio; GEC, gene expression classifier; GSC, gene sequencing classifier; NLR, negative likelimehood ratio; No, number; PLR, positive likelihood ratio; SENS, sensitivity; SPEC, specificity.

#### Diagnostic Performance of Afirma GSC

**Figure 4:**
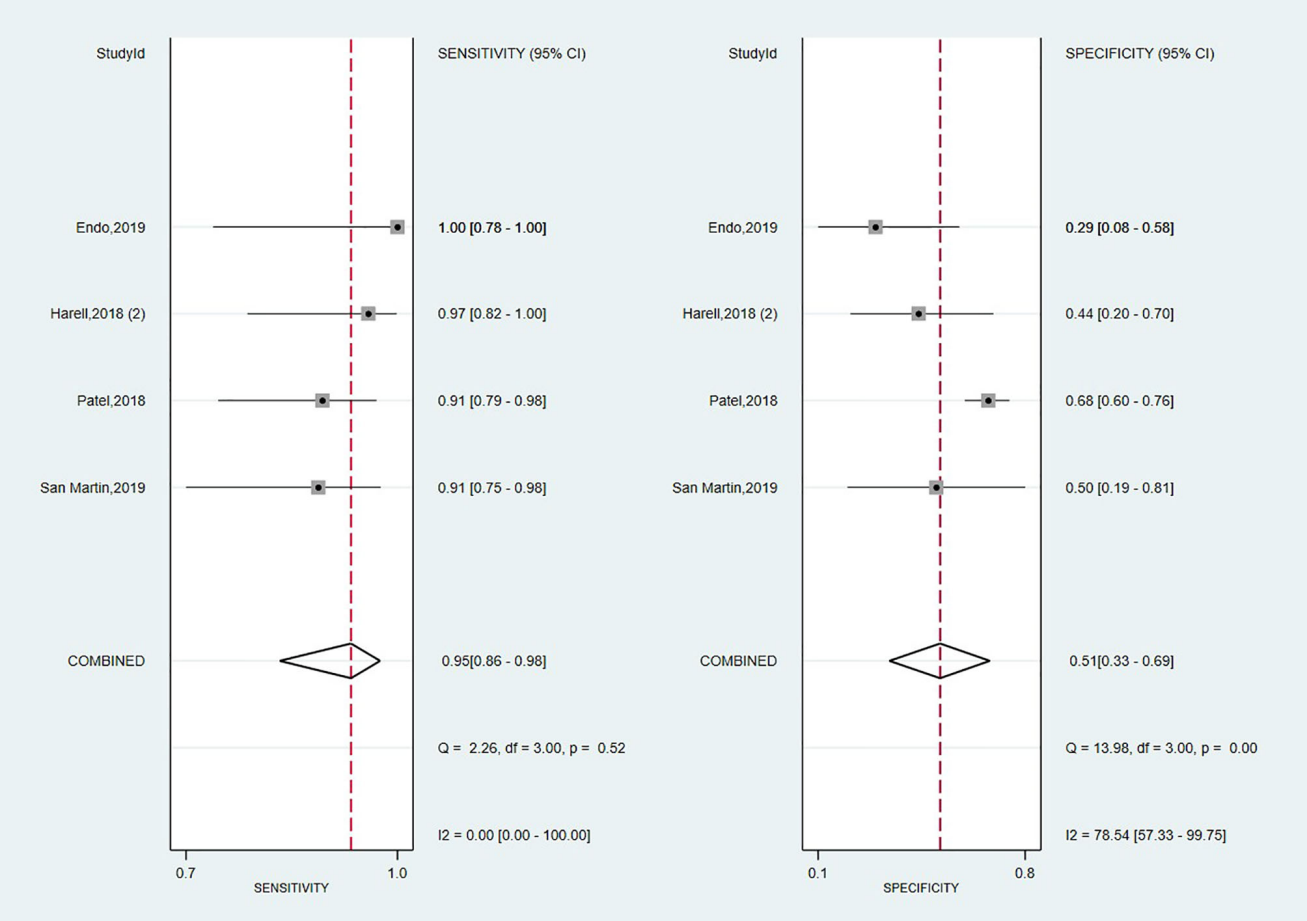
Forest plot of sensitivity and specificity for Afirma GSC panel.

Looking foreword at the studies exploring Afirma GSC panel (16, 17, 57, 70), the forest plot displayed in **Figure 4** showed low heterogeneity with a large CI among studies regarding SE (I^2^ = 0%, 95% CI: 0 to 100) and high heterogeneity regarding SP (I^2^ = 79% 95% CI: 57 to 100). Thus, we applied the random effect model for the cumulative values. The results showed an overall SE of 0.95 (95% CI: 0.86 to 0.98), SP of 0.51 (95% CI: 0.33 to 0.69), PPV of 0.60 (0.52–0.68) and a NPV of 0.91 (0.80–0.68). The overall BCR for Afirma GSC was 73%.

The AUC value from the SROC curve, displayed in **Supplementary Figure 2**, was 0.90 (95% CI: 0.87 to 0.98), indicating an excellent overall detection of the Afirma GSC panel. Also, Afirma GSC proved a modest magnitude of change in test-positive cases based on PLR of 1.9 (95% CI: 1.3–2.8) but stronger evidence to change the probability in test-negative cases according to NLR of 0.11 (95% CI: 0.04–0.27), as seen in **Table 2**. Besides, the DOR of 18 showed a lower value than Thyroseq v3 but was associated with a narrower 95% CI (6–50, 103, 104). However, as we had only four studies on which to rely on our estimation, we precluded the sensitivity analyses with the impact of NIFTPs reclassification, TBSRTC categories, and declared conflicts of interest in this panel’s case.

#### Diagnostic Performance of Thyroseq v2

**Figure 5:**
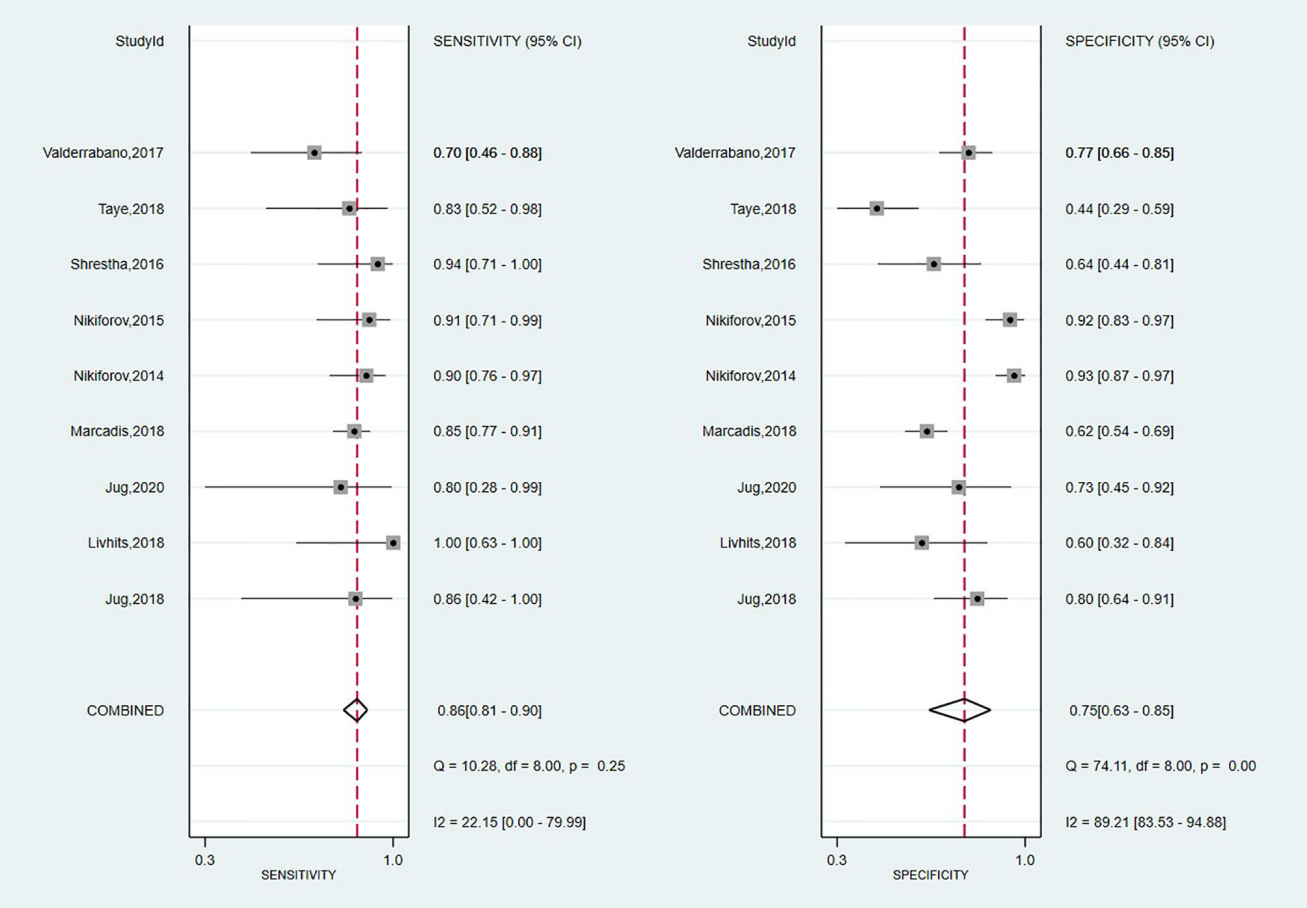
Forest plot of sensitivity and specificity for Thyroseq v2 panel.

A total of nine studies have looked at the diagnostic accuracy of Thyroseq v2 (11, 15, 58, 59, 61, 62, 71, 73, 75). The forest plot is shown in **Figure 5**, with an overall value for SE of 0.86 (95% CI: 0.81 to 0.90) and SP of 0.75 (95% CI: 0.63 to 0.85). A low heterogeneity for SE (I^2^ = 22%, 95% CI: 0 to 80) and high for SP (I^2^ = 89% 95% CI: 84 to 95) stands out. The area under the SROC curve was 0.88 (95% CI: 0.85–0.90; **Supplementary Figure 3**). Thyroseq v2 demonstrated a PPV of 0.51 (95% CI: 0.41–0.60), a NPV of 0.95 (95% CI: 0.85–1.00) and a BCR of 73%.

The Fagan plot (**Supplementary Figure 4**) showed that in the low suspicion of malignancy scenario (25%), a PLR of 3.5 increases the post-test probability for a positive test result to 54%, whereas an NLR of 0.11 reduced the post-test probability to 6% for a negative test result. On the other hand, given a pre-test probability of 75% in the high suspicion scenario, a positive posterior probability of 91% could be considered to diagnose TC and the post-test probability was 35% for a negative test result. Also, we computed the DOR, which showed a similar value to Afirma GSC but a narrower 95% CI [19; (9–42)].

##### Impact of Bethesda Categories and Conflict of Interests on Thyroseq v2 Diagnostic Performance

Looking specifically at the Bethesda III category TNs, the four studies included (15, 71, 73, 75) showed no changed results for SE (0.85; 95% CI: 0.57–0.96), a mild decrease in SP (0.70; 95% CI: 0.46–0.87) and AUC (0.85; 95% CI: 0.82–0.88). Regarding the Bethesda IV category, we have noticed an insignificant decrease in SE (0.73; 95% CI: 0.40–0.91) and AUC (0.83; 95% CI: 0.79–0.88) accompanied by a large heterogeneity of the results. Due to lack of data, we did not perform a separate Bethesda V nodules analysis (see **Supplementary Figures 5–8**).

The SP across studies that declared no conflicts of interest or industry sponsorship (58, 59, 61, 62, 73) was lower (0.60, 95% CI: 0.51 to 0.75) compared to overall result for Thyroseq 2, decreasing consequently the heterogeneity around SP to I^2^ = 70.9%, 95% CI: 43.9 to 98.0. However, AUC decreased insignificantly to 0.86 (95% CI: 0.83 to 0.89) when performed SROC curve for this subgroup of studies (**Supplementary Figures 9, 10**).

It has not been possible to compute a sensitivity analysis for repeated FNAs due to the limited number of studies evaluating Thyroseq v2.

#### Diagnostic Performance of Afirma GEC

**Figure 6:**
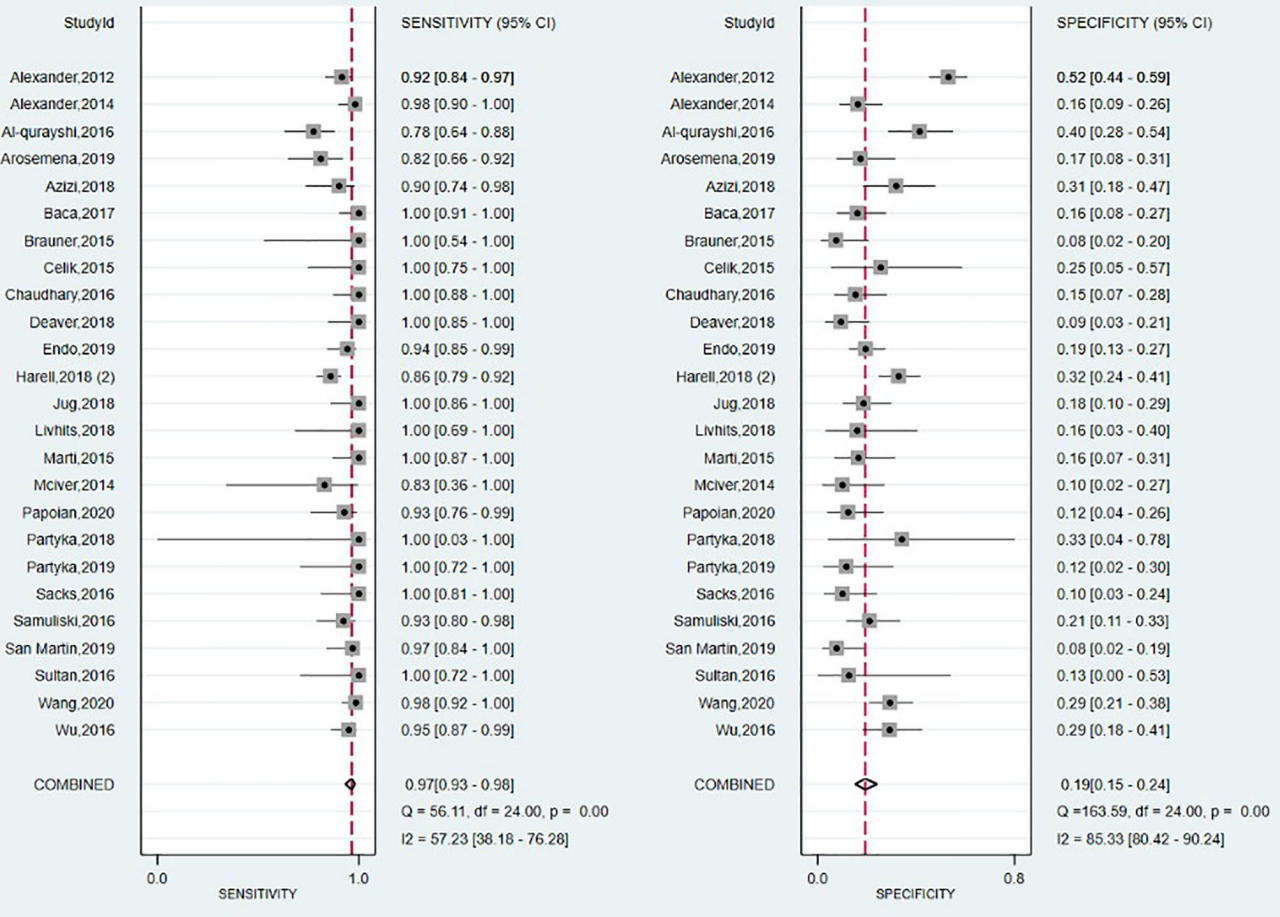
Forest plot of sensitivity and specificity for Afirma GEC panel.

The forest plot summarizing the data from the 25 studies involving Afirma GEC assay in diagnosing TC is shown in **Figure 6**. As high heterogeneity between studies in SE and SP data (I2 = 57%, 95% CI: 38 to 76; respectively I^2^ = 85%, 95% CI: 80 to 90) was observed, the random effect size was applied for computing the meta-analysis. The overall SE and SP were 0.97 (95% CI: 0.93 to 0.98) and 0.19 (95% CI: 0.15 to 0.24), and PPV and NPV were 0.39 (95% CI: 0.37–0.40) and 0.91 (95% CI: 0.88–0.93), respectively. Afirma GEC showed the lowest DOR of 7, in conjunction with a narrow 95% CI which is in the range of 3 to 13, and a BCR of 42%.

The SROC curve presented in **Supplementary Figure 11**, and the corresponding value for the AUC, was 0.61 (95% CI: 0.56 to 0.65), indicating a low overall detection.

However, to better understand the overall detection efficacy, we have further performed a sensitivity analysis series. Excluding those studies (8, 48, 67, 76) pinpointed by the bivariate box plot (see **Supplementary Figure 12**) as outliers we retrieved a SE of 0.97 (95% CI: 0.94 to 0.98), SP of 0.17 (95% CI: 0.14 to 0.20) and an AUC of 0.47 (95% CI: 0.42 to 0.51), as presented in **Supplementary Figures 13, 14**.

In Fagan’s nomogram low suspicion of TC scenario (25%), the post-test probability for a positive test result was 28%, whereas an NLR of 0.11 reduced the post-test probability to 6% for a negative test result (**Supplementary Figure 15**). On the other hand, given a pre-test probability of 75% in the high suspicion scenario, a positive posterior probability increases to 78%, and the negative posterior probability decreases to 35%, respectively.

Considering the small study effects, the Deeks’ funnel plot for the 25 studies included in our meta-analysis indicated no evidence of publication bias (p = 0.19 for Deeks’ funnel plot asymmetry test; see **Supplementary Figure 16**).

##### Impact of Bethesda Categories, Repeated FNA, and Conflict of Interests on Afirma GEC Diagnostic Performance

Looking specifically at the Bethesda III category TNs, the eleven studies included showed a slightly decreased overall SE (0.94, 95% CI: 0.88 to 0.97) and an increased overall SP (0.23, 95% CI: 0.14 to 0.35) of Afirma GEC (8, 17, 49, 51, 53, 54, 56, 64, 66, 70, 77). Moreover, the heterogeneity between studies was dampened to 33% (95% CI: 0 to 81) in the case of SE (**Supplementary Figure 17**). The AUC from the SROC curve was 0.83 (95% CI: 0.80 to 0.86), indicating that Afirma GEC has good accuracy when used in AUS/FLUS patients (**Supplementary Figure 18**). Concerning TNs of Bethesda IV category based on eight studies (8, 17, 53, 54, 56, 64, 70, 77), we have found an AUC of 0.95 (95%CI: 0.92 to 0.96), an overall SE of 0.95 (95% CI: 0.89 to 0.98) and SP of 0.20 (95% CI: 0.10 to 0.35) with low heterogeneity around SE (*I* ^2^ = 1.97% 95% CI: 0 to 100) and high heterogeneity around SP (*I* ^2^ = 89% 95% CI: 0 to 100) (**Supplementary Figures 19, 20**).

Also, when we have looked at studies evaluating Afirma GEC, that performed a repeat FNA to confirm indeterminate cytology (50–52, 54, 58, 70, 72), we noticed an increase in AUC from 0.61 (95% CI: 0.56 to 0.65) to 0.83 (95% CI: 0.79 to 0.86), even though SE and SP were slightly changed (**Supplementary Figures 21, 22**).

A separate analysis was performed considering just studies that declared no conflicts of interest or industry sponsorship (17, 49, 50, 53, 54, 58, 61, 63, 64, 66–68, 70, 72, 76). Thus, we noted a decrease in Afirma GEC’s performance by the decline of AUC from 0.61 (95% CI: 0.56 to 0.5) to 0.43 (95% CI: 0.39 to 0.48), instead not affecting SE and SP meaningfully, as seen in **Supplementary Figures 23, 24**.

#### Impact of NIFTP Cases Reclassification on Afirma GEC and Thyroseq v2 Diagnostic Performance

To investigate the impact of revised nomenclature of encapsulated FVPTC and NIFTP reclassification on the molecular test performance we included into analysis only studies (17, 40, 51, 56, 58, 61, 68, 72) where the NIFTPs and their test results were reported. Regarding Afirma GEC, we have observed a slight increase in SE (0.98, 95% CI: 0.85 to 1.00) and a decreased overall SP of 0.14 (95% CI: 0.11 to 0.19). The corresponding AUC was 0.25 (95% CI: 21 to 29; **Supplementary Figures 25, 26**).

Regarding the scenario for Thyroseq v2 where NIFTPs are assumed as nonmalignant histology, the SE (0.82, 95% CI: 0.68 to 0.91) and SP (0.60, 95%CI: 0.49 to 0.69), as well as AUC (0.76, 95% CI: 0.72 to 0.80) decreased compared to primary results. Moreover, this analysis importantly decreased the heterogeneity around SE (I2 = 70.9%, 95% CI: 0.0 to 90.2) and SP (I^2^ = 75.1%, 95% CI: 54.8 to 95.4; **Supplementary Figures 27, 28**).

We could not perform analogous analysis for the rest of the molecular tests due to the limited number of studies.

## Discussion

Molecular tests are increasingly used as auxiliary diagnostic tools aimed to help avoid both diagnostic and completion surgeries in cytologically ITNs. Previous panels, Thyroseq v2 and Afirma GEC, have proven shortcomings in malignancy detection performance. The present study is the first one to provide a comprehensive analysis of the novel molecular tests, Thyroseq v3, Afirma GSC, multiplatform, and miRNA-based assays for the malignancy assessment in ITNs, to the best of our knowledge.

According to the predominant ability to exclude or confirm a malignancy, the molecular panels are classified as “rule-in” or “rule-out” tests (105). Vargas-Salas et al. showed that, considering the cancer prevalence range of 20–40%, a robust “rule-out” test would require an NPV of at least 94% and a minimum SE of 90%, while for a desirable test to predict or “rule-in” malignancy, an optimal standard would be a PPV of at least 60% and an SP above 80%. These parameters are associated with both, optimal clinical accuracy and clinical effectiveness (105). A “rule out” test will perform better in a low-risk TN at US or in a cytologic category of low cancer frequency such as Bethesda III or IV category (106). Sonographically high-risk TNs or categories of higher cancer frequency such as in Bethesda V would benefit more from a “rule-in” test, in which case a positive test result would decrease the risk of completion surgery (106).

Our results suggest that ThyroSeq v3 shows excellent diagnostic accuracy compared with its prior iteration based on an AUC of 0.95. Also, Thyroseq v3 showed the lowest NLR of 0.02, making it the most accurate test to exclude malignancy. However, the SE and NLR improved at the expense of decreasing SP and PLR, declining the ability to confirm malignancy. The validity of these results is still questionable, considering the small number of studies evaluating this panel and data instability due to outliners; hence, the ability of Thyroseq v3 to “rule-in” malignancy should be confirmed in future studies. Besides, in theoretical modeling, Thyroseq v3 was slightly more cost-effective than Afirma GSC and considerably more cost-effective than diagnostic lobectomy (107).

Afirma GSC succeeded partially to reach its original objective to increase the “rule-in” properties of GEC, given the modest increase in SP and PLR. However, GSC managed to improve substantially the NLR to 0.11 and BCR from 42 to 73%, making GSC even a better “rule-out” test compared with its front-runner. These findings are in line with previous literature results, which showed a significant increase in BCR (65.3% *vs* 43.8%) compared to that of Afirma GEC (108). The overall performance of Afirma GSC is considerably improved, given the increase in the AUC to 0.90 and the DOR to 18. GSC could, therefore, be an excellent “rule out” test. However, its “rule-in” properties have not been confirmed, and thereby, the management of cases with suspicious tests should be made, including other clinical, US, and cytological characteristics.

Based on the pooled results from nine studies, Thyroseq v2 shows a good overall performance, owing to the AUC of 0.88 and DOR of 19, similar to Afirma GSC. Also, Thyroseq v2 showed the highest PLR, making it the first option from those available to confirm malignancy. However, the PLR of 3.5 examined separately can produce a small shift in malignancy probability. Therefore, Thyroseq v2 strength continues to be in its “rule-out” features, considering the NLR of 0.18, which can generate a shift in post-test probability in the low suspicion scenario from 25 to 6%. When separate analyses by TBSRTC were computed, a slight decrease in SE and increase in SP among Bethesda IV compared to Bethesda III TNs was noticed, thus, suggesting that Thyroseq v2 could be more effective in rule-in malignancy in TNs with higher pre-test prevalence of malignancy. The industry sponsorship and conflicts of interest did not affect the results except for a slight decrease in SP (42). However, controversies exist regarding the clinical utility of this molecular test, especially due to the lack of decrease in the surgery rate along with the additional cost of Thyroseq v2 that can increase the overall cost of care of patients with ITNs (13, 109). Moreover, the introduction of ThyroSeq v2 resulted in a shift toward indeterminate cytology results (13).

Regarding Afirma GEC, our analysis based on the pooled results across 25 articles showed unsatisfactory overall diagnostic performance (AUC 0.60) and poor ability to confirm malignancy given the PLR of 1.2. However, when patients were segregated by TBSRTC categories, Afirma GEC reached an AUC of 0.83 for AUS/FLUS and 0.95 for FN/SFN. Also, performing the Afirma GEC test in persistently indeterminate TNs could increase the AUC of GEC. In this regard, several studies claim that AUS/FLUS and SUSP nodules are reclassified after the repeat FNA in a proportion from 10% to 40%, usually into a benign category (110–112), hence, affecting the accuracy of the results. It seems that industry sponsorship and conflicts of interest could affect the results for Afirma GEC accuracy. Therefore, based on the optimal NLR, Afirma could be helpful as a “rule-out” test, especially in Bethesda III and IV lesions. It might help in predicting benign TNs in cytologic categories with low cancer frequency, in low-risk TNs at US, or when clinical follow-up is recommended instead of diagnostic surgery.

Recently, Liu et al. performed a meta-analysis assessing the diagnostic performance of Afirma GEC. Similar to our results, they showed that Afirma GEC has a relatively high SE of 95.5%, but a low SP of 22.1% and DOR of 5.25, concluding that the outcome for over half of the nodules with GEC-suspicious is still uncertain, which limits its use in clinical practice (42). Interestingly, the routine use of Afirma GEC in clinical practice seemed to increase the incidence of indeterminate FNA diagnoses, whereas the incidence of benign diagnoses significantly decreased. These results suggest that Afirma GEC may shift FNA interpretation toward Bethesda III/IV, in which molecular testing is used. Moreover, the surgery rate did not appear to change in an institutional retrospective study, raising uncertainty regarding the benefits of this molecular assay in risk stratification (69). Other authors have shown overtreatment among patients whose management was decided following this test result (113).

Due to the limited number of studies, we could not compute separate analyses for Interpace’s multiplatform tests, RosettaXG Reveal, and miRInform in the MIDAS framework, which requires a Gaussian quadrature (114). For this reason, we have reported the abovementioned molecular panels SE and SP range across the studies as preliminary evidence. In this regard, The Interpace multiplatform approach provided an optimal SE, across studies but a slightly decreased SP compared to that claimed by its predecessor miRInform. Finally, the recently introduced Rosetta GX Reveal reported an optimal diagnostic accuracy. However, there is a severe concern about the instability of the results, especially the Interpace platform which combines two separate panels, and we need future studies to validate these diagnostic tests and their clinical utility.

The secondary objective of the research was to investigate the impact of revised nomenclature of encapsulated FVPTC and NIFTP reclassification on the aforementioned molecular test performance (28). Our findings support that Afirma GEC and Thyroseq v2 performance outcomes were affected by NIFTP reclassification, due to the increase in FPs rate. As would be expected from a “rule-out” test, Afirma GEC’s Se and Sp were not significantly affected, even though AUC markedly dropped. However, as regards Thyroseq v2, a more critical change, especially in Sp was noticed. Reflecting a similar trend to the present results, a recent analysis by Sahli et al. reported an insignificant decrease in Se and Sp for Afirma GEC and a more critical change in the diagnostic performance of Thyroseq v2 after the addition of the new diagnostic entity (38). They also found a decrease in PPV from 47 to 38% for Afirma GEC and from 83 to 29% for Thyroseq v2, respectively (38).

This reclassification of NIFTP lesions from malignant to premalignant has an important impact concerning the diagnostic performance of molecular tests. It was described previously that Afirma GEC and Thyroseq v2 can detect the genetic alterations, such as RAS gene mutations, THADA fusions, PPARc-PAX8 fusions, and BRAFK601 mutation (28, 115). Due to the presence of RAS mutations in a significant number of NIFTPs (116), molecular panels will mark NIFTP as “suspicious” for malignancy (115). Moreover, because of the wide variability of genetic mutations among benign thyroid lesions, cautious interpretation of current genetic testing results (117) and recalibration to appropriately account for the NIFTPs is required.

A potential limitation of this review and meta-analysis was that the analyzed diagnostic tests could not be compared and ranked due to the limited number of studies with direct head-to-head comparisons. Second, only patients with surgical pathology were considered and, therefore, excluding many benign nodules by molecular testing managed conservatively. The rationale behind this decision is the inferior reliability of clinical and sonographic follow-up compared to that of histopathology, which is considered the diagnostic gold standard, especially because, in most of the studies, the mean follow-up was less than 2 years. Moreover, statistically, the evidence comparing an assay with the gold standard (i.e., surgery) as well as with other conservative methods (i.e., sonographic follow-up) should be treated as different analyses, because mixing the results could lead to biased results in pairwise meta-analyses (118). Thus, the decision to proceed otherwise would have led to differential reference bias. Third, final pathology was unavailable, especially for those with a benign test result, due to the choice to undergo conservative management. Fourth, all the studies were performed in the USA population, thus raising some concerns regarding the extrapolation of the results to the rest of the world. Finally, an overall unclear methodological quality of the included studies could have led to inaccurate assumptions.

In most TCs, genetic alterations are mutually exclusive events (119). Some mutations, like BRAF V600E and TERT, are highly specific, showing almost a 100% risk of PTC (120, 121). However, the impact of RAS mutations or PAX8/PPARγ rearrangements is still evolving since they show a considerable overlap among different morphological entities. RAS mutations, RET/PTC, and PAX8/PPARγ rearrangements were detected in up to 48, 68, and 55% of all benign nodules, respectively, while some malignant lesions showed no mutations at all (122). Variable number and types of mutations among benign nodules may explain the low Sp and PPV of Afirma GEC (122) and may also challenge the reported PPV of Thyroseq V2 (14). Newer products, Afirma GSC and Thyroseq v3, begun to address the challenges discussed above (122). As experience accumulates, we will gain a deeper insight into how well they mitigate the challenges addressed herein.

The development of new biomarkers in TCs will most likely lead to enhanced versions of current tests or the development of new ones. The ultimate goal of each molecular testing of cytological samples from ITNs is to add evidence in support or against the need for surgical treatment and the extent of surgery, to achieve the individual patient’s best outcome. Thus, it will be necessary to determine whether negative test results indeed decrease the number of unnecessary surgeries and a positive result reduces the rate of completion surgeries. Besides, new hopes are directed towards the updated Afirma GSC and XA reports. The impact of Afirma XA could extend beyond informing upon the risk of cancer when the test result is negative or positive, for a specific genomic alteration. It gives potential insights into the molecular analysis of the FNA specimens claiming to inform about the associated neoplasm types, prognostics, identification of molecular targets for systemic therapy, and the recognition of potential hereditary syndromes (18, 20). Future evidence is needed to validate the Afirma XA real-word performance.

## Conclusions

Summarizing all the data obtained in this comprehensive meta-analysis, the conclusion that can be drawn is that there is no perfect molecular panel at the current time to discriminate malignancy in ITNs. However, each of the tests above has its strong points and can be used in particular situations. Our results suggest that Thyroseq v3 substantiate the best overall diagnostic performance, followed by Afirma GSC and Thyroseq v2, which were similar in terms of AUC and DOR. In terms of “rule-out” performance, Thyroseq v3 showed the most noticeable results, being able to generate a large shift in cancer probability of a negative test result. However, optimal results to exclude malignancy can be achieved with Afirma GSC but also with previous tests, no longer available, Afirma GEC, and Thyroseq v2. If considering the “rule-in” properties, the recently developed Thyroseq v3 and Afirma GSC failed to achieve a higher performance to confirm a malignancy, being surpassed by Thyroseq v2. Secondly, MPTX and RosettaGX show excellent preliminary results, and future studies are needed to validate them. The quality of evidence seems to be higher for Thyroseq v3, notwithstanding the limited number of studies.

## Data Availability Statement

The original contributions presented in the study are included in the article/**Supplementary Material**. Further inquiries can be directed to the corresponding authors.

## Author Contributions

CAS and VL conceived and designed the research, drafted the protocol, abstracted the total data from the included articles, and participated in writing the manuscript. RDG and AD conducted the statistical analysis/meta-analysis. SS, BAN, and CEG participated in the search, screening, and analysis of the literature. HS supervised the research, contributed in project administration, and critically revised the manuscript. All authors contributed to the article and approved the submitted version.

## Funding

This work was supported by the Romanian Ministry of Education and Research, CCCDI-UEFISCDI, project code PN-III-P2-2.1-PED2019-2536 within PNCDI III.

## Conflict of Interest

The authors declare that the research was conducted in the absence of any commercial or financial relationships that could be construed as a potential conflict of interest.
