# Supplementary material for: Thyroseq v3, Afirma GSC, and microRNA Panels Versus Previous Molecular Tests in the Preoperative Diagnosis of Indeterminate Thyroid Nodules: A Systematic Review and Meta-Analysis
Source: Front Endocrinol (Lausanne). 2021 May 13;12:649522. doi: 10.3389/fendo.2021.649522 (PMC8155618; doi:10.3389/fendo.2021.649522)
Supplement: Supplementary file 1 [file DataSheet_1.docx]

Supplementary Material

# Supplementary Figures and Tables

## Supplementary Figures


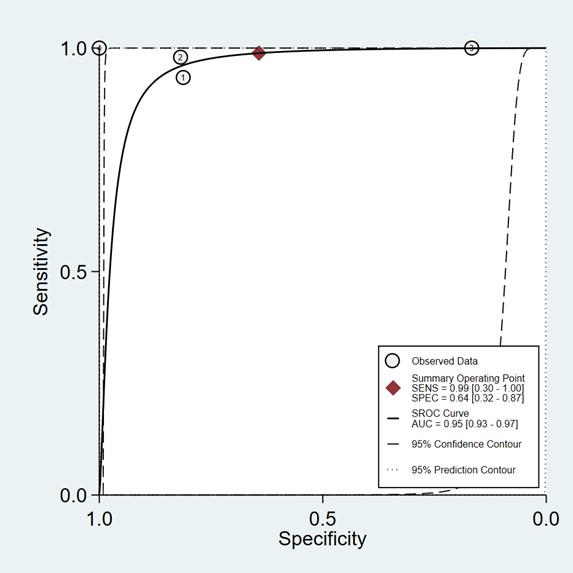


**Supplementary Figure 1**: Summary receiver operating curve for Thyroseq v3 panel


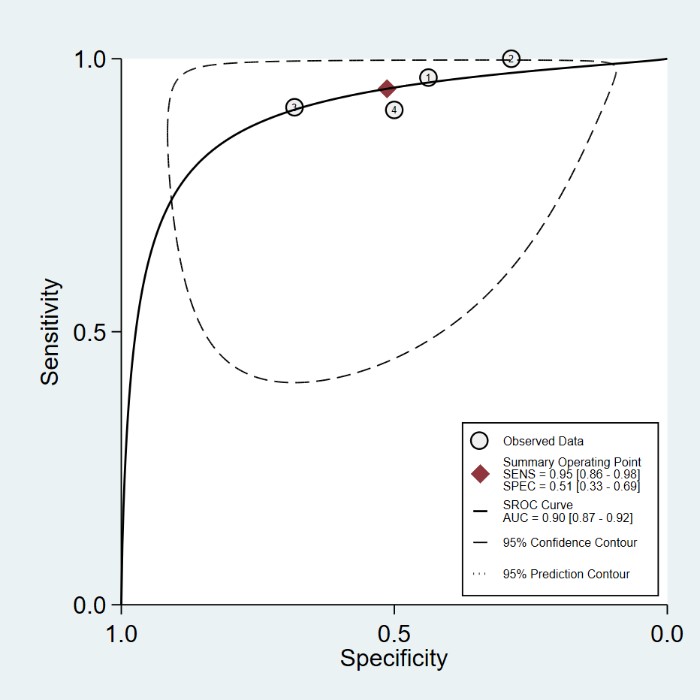


**Supplementary Figure 2**: Summary receiver operating curve for Afirma GSC


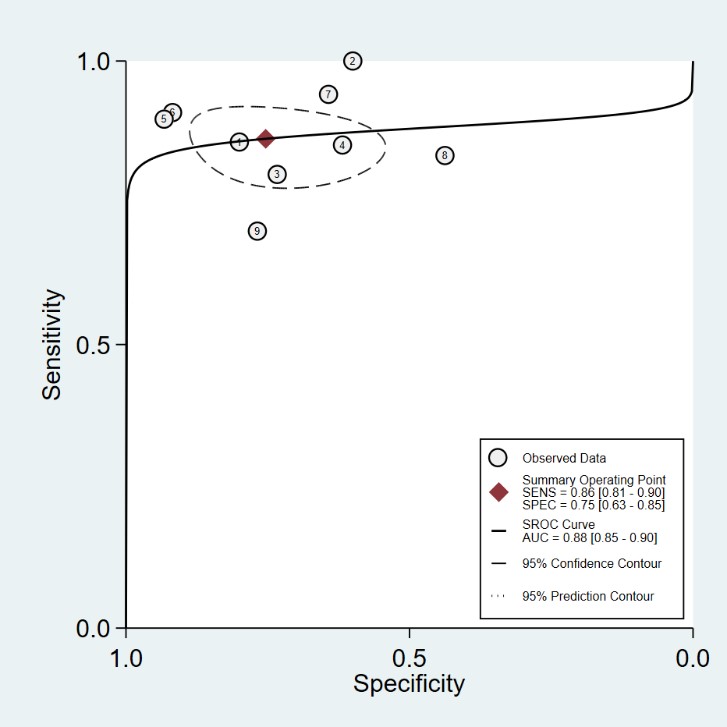


**Supplementary Figure 3**: Summary receiver operating curve for Thyroseq 2


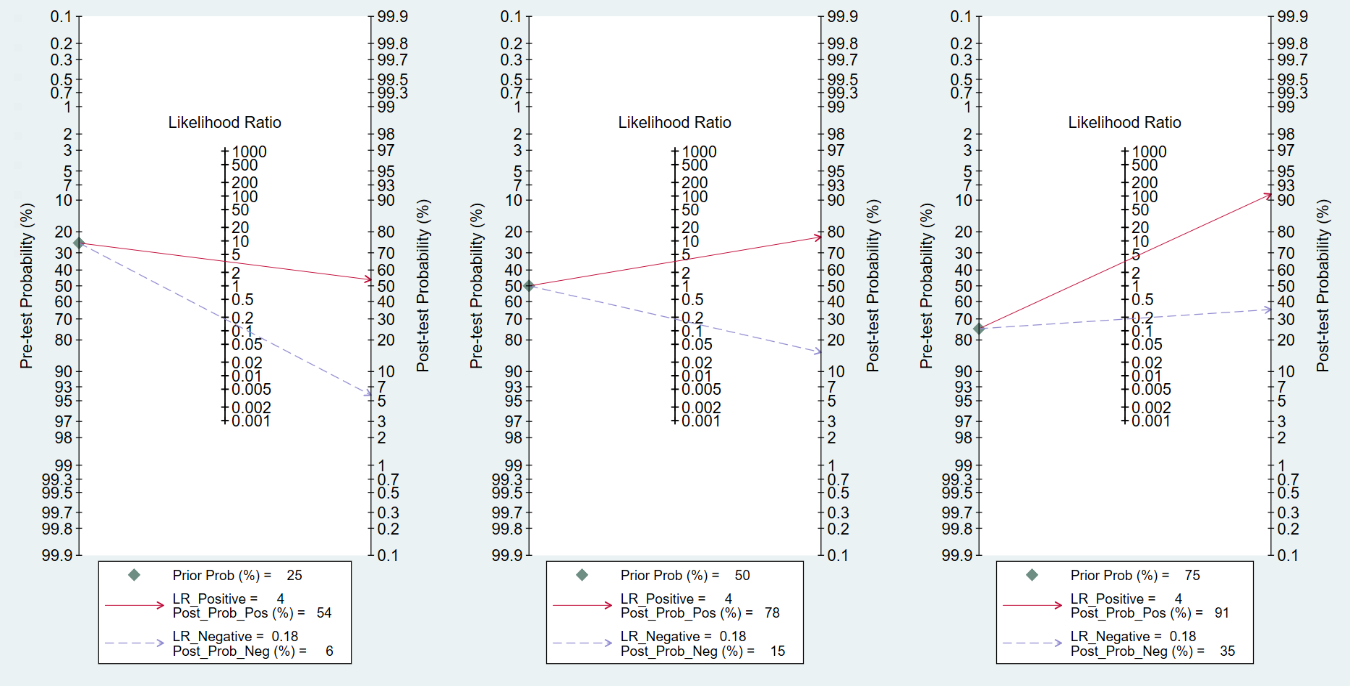


Supplementary Figure 4: Fagan’s nomogram for Thyroseq 2


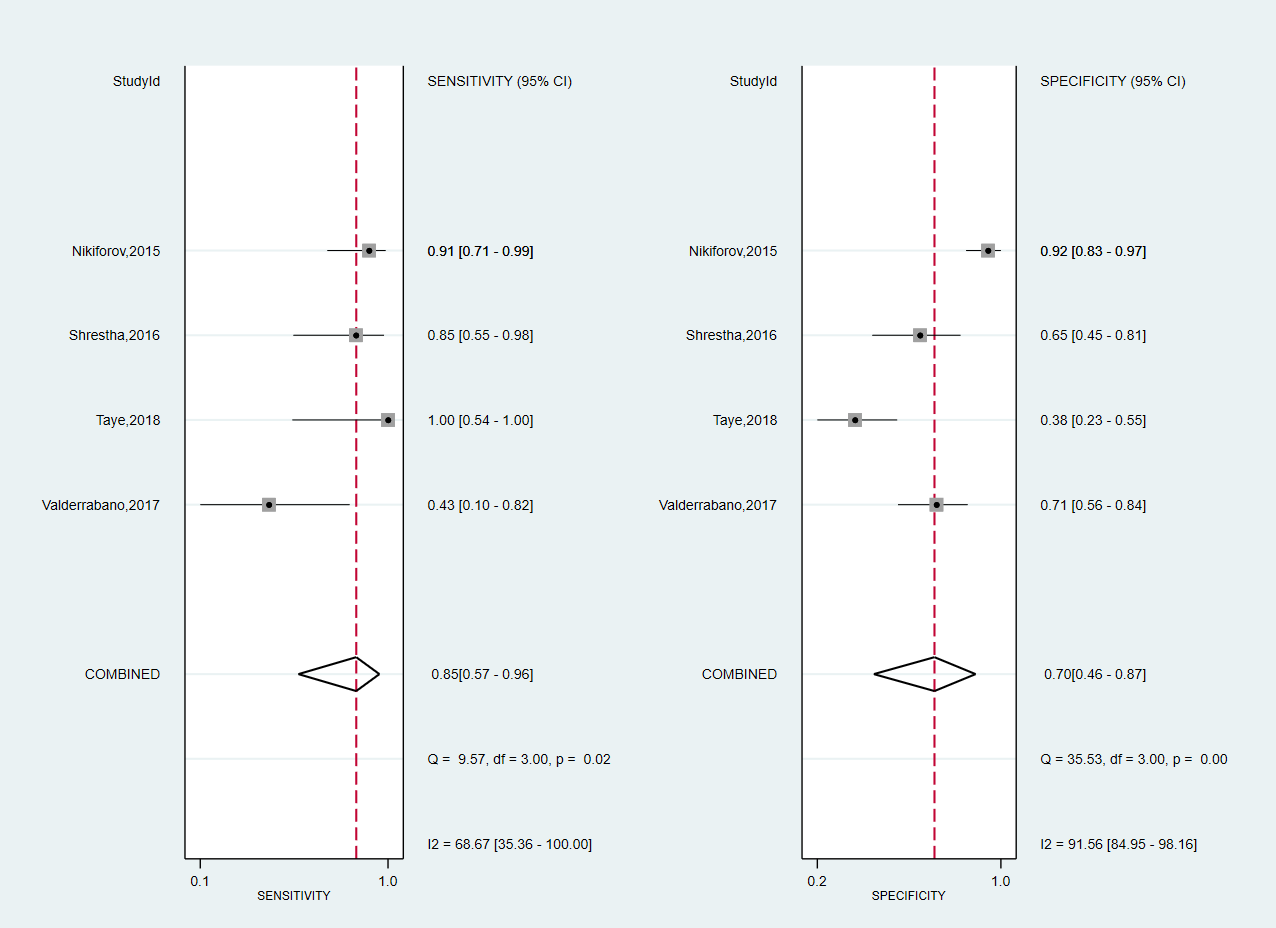


Supplementary Figure 5: Forest plot for Thyroseq 2 overall sensitivity and specificity in Bethesda III nodules


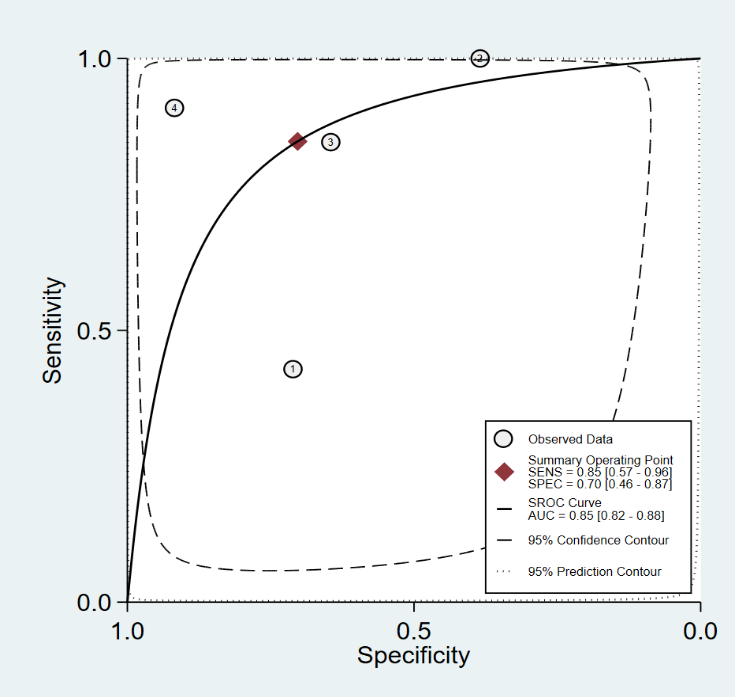


Supplementary Figure 6: SHROC curve for Thyroseq 2 overall sensitivity and specificity in Bethesda III nodules


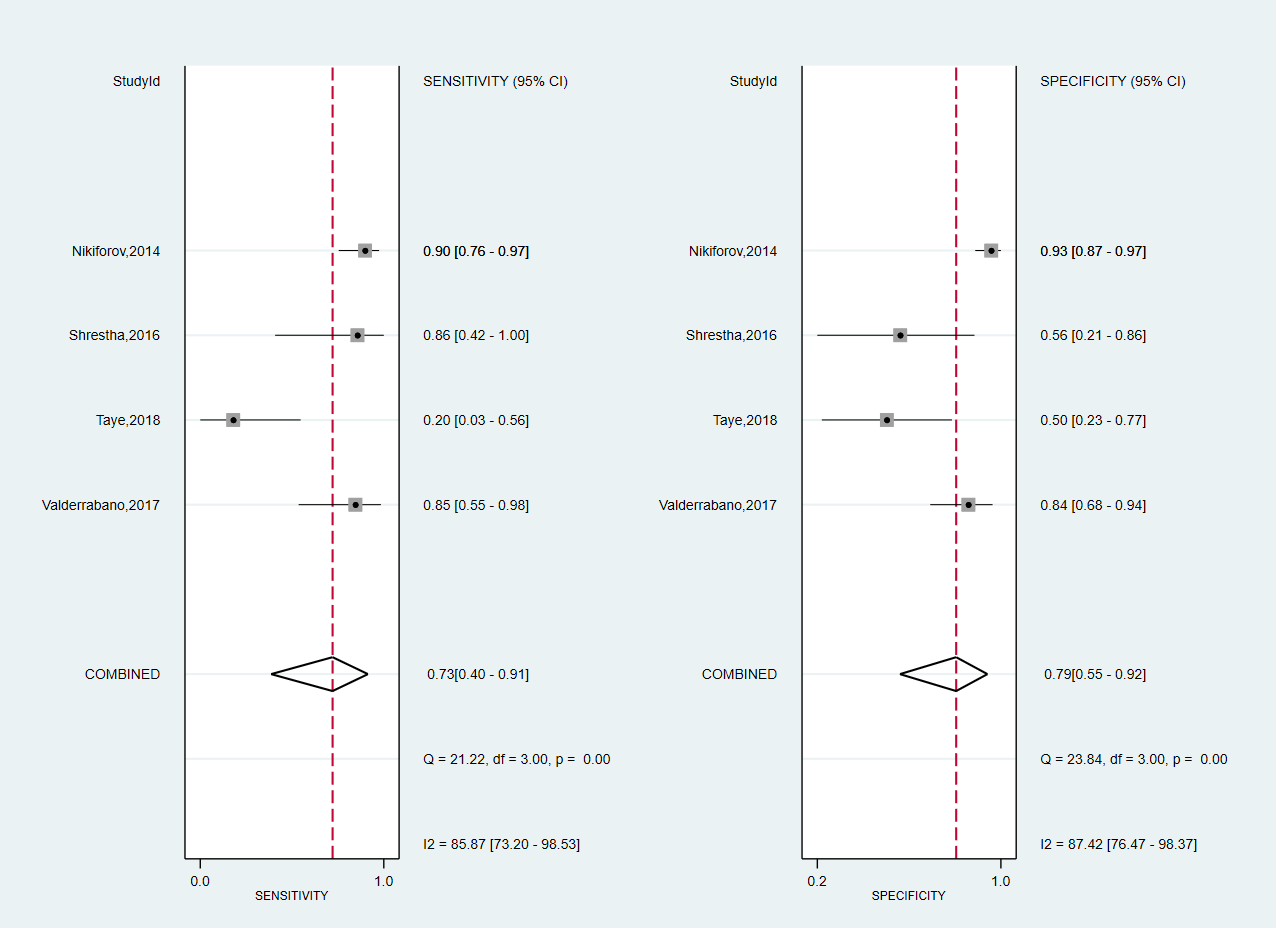


Supplementary Figure 7: Forest plot for Thyroseq 2 overall sensitivity and specificity in Bethesda IV nodules


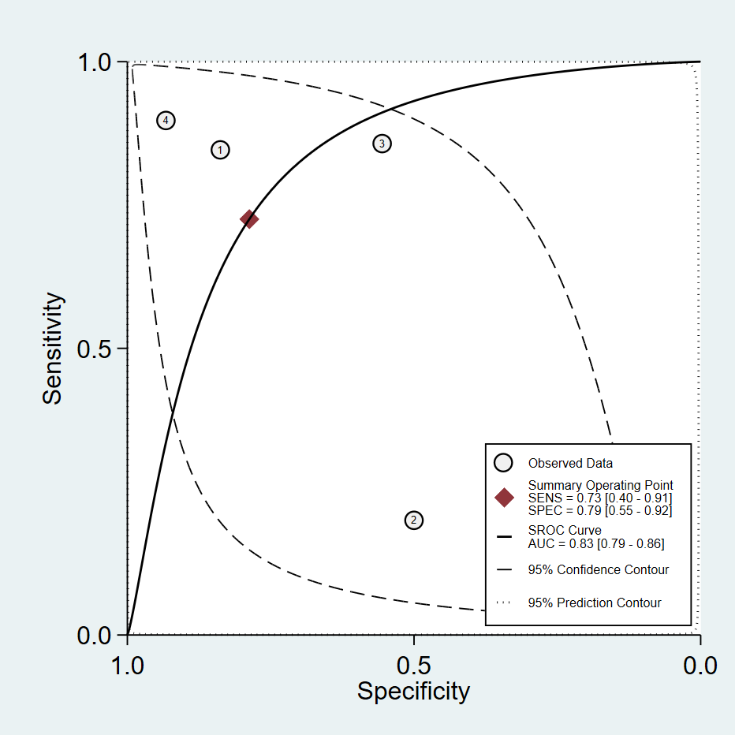


Supplementary Figure 8: SHROC curve for Thyroseq 2 overall sensitivity and specificity in Bethesda IV nodules


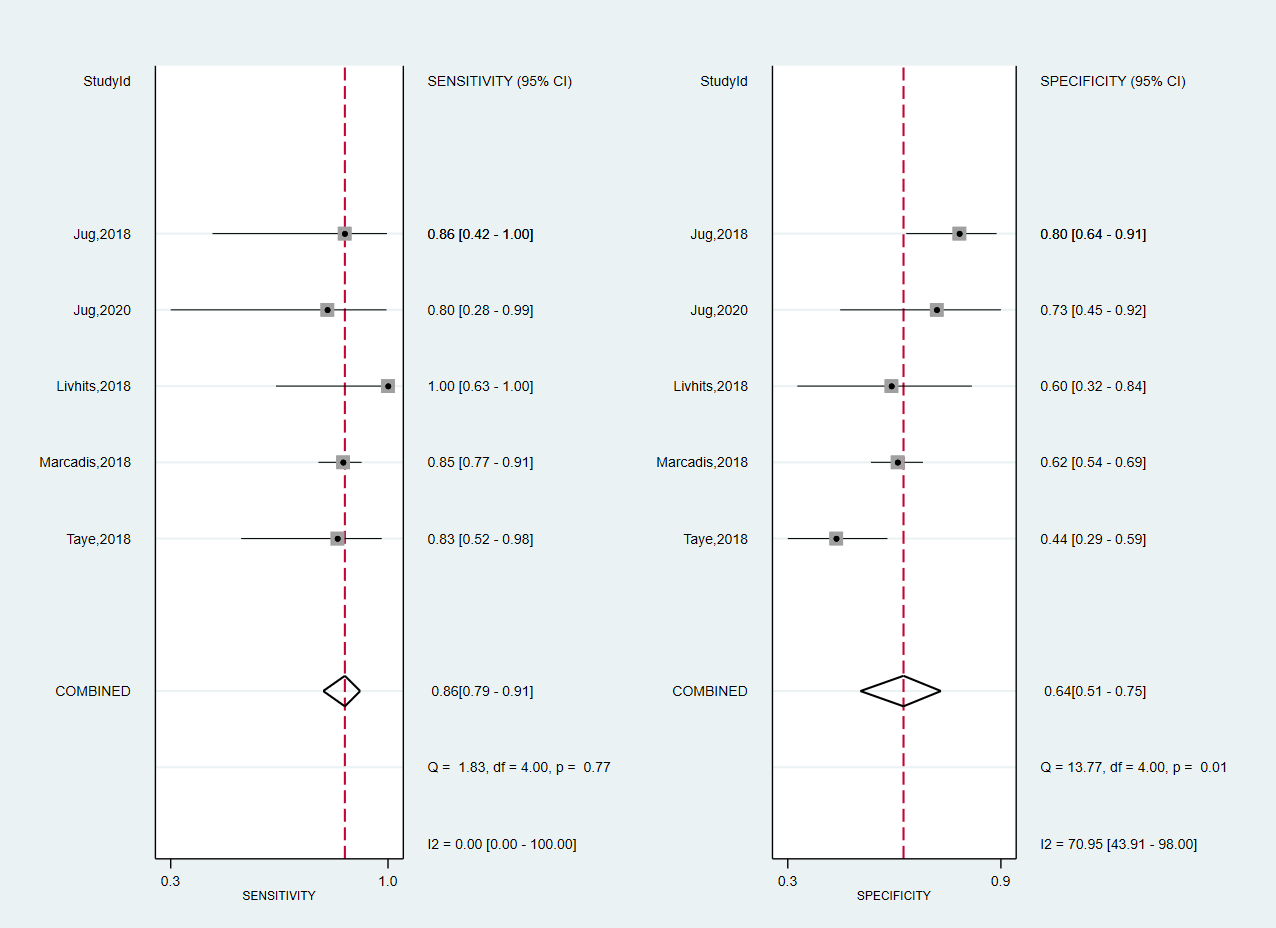


Supplementary Figure 9: Forest plot for Thyroseq 2 overall sensitivity and specificity including studies without declared conflicts of interests


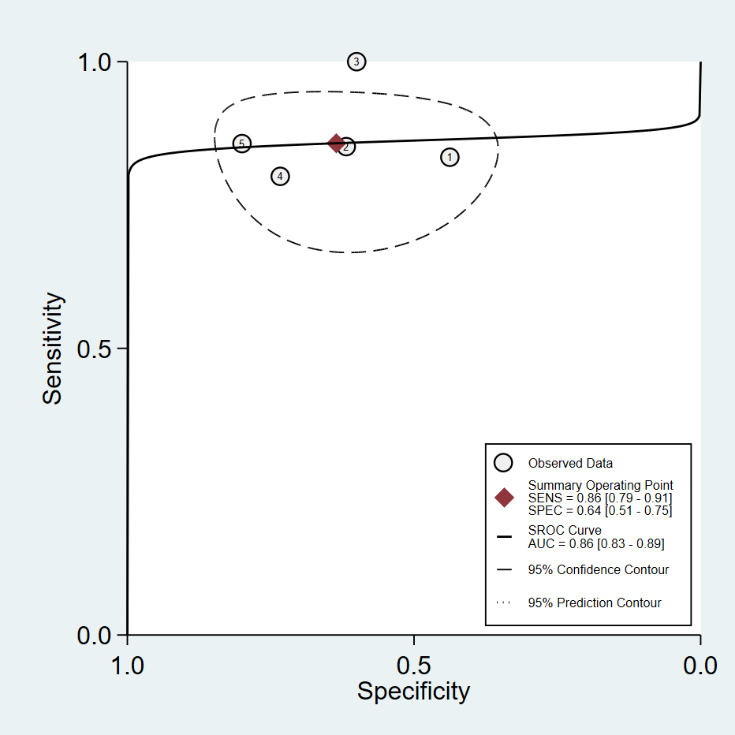


Supplementary Figure 10: SHROC curve for Thyroseq 2 overall sensitivity and specificity including studies without declared conflicts of interests


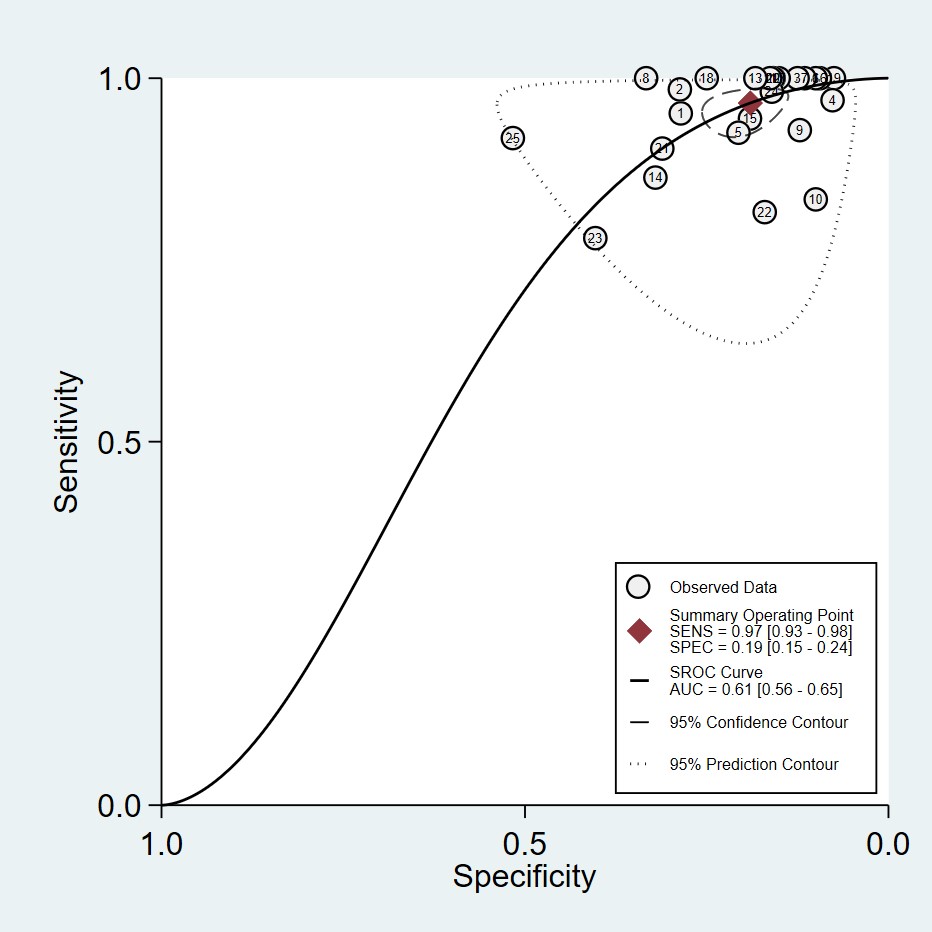


**Supplementary Figure 11**: Summary receiver operating curve for Afirma GEC panel


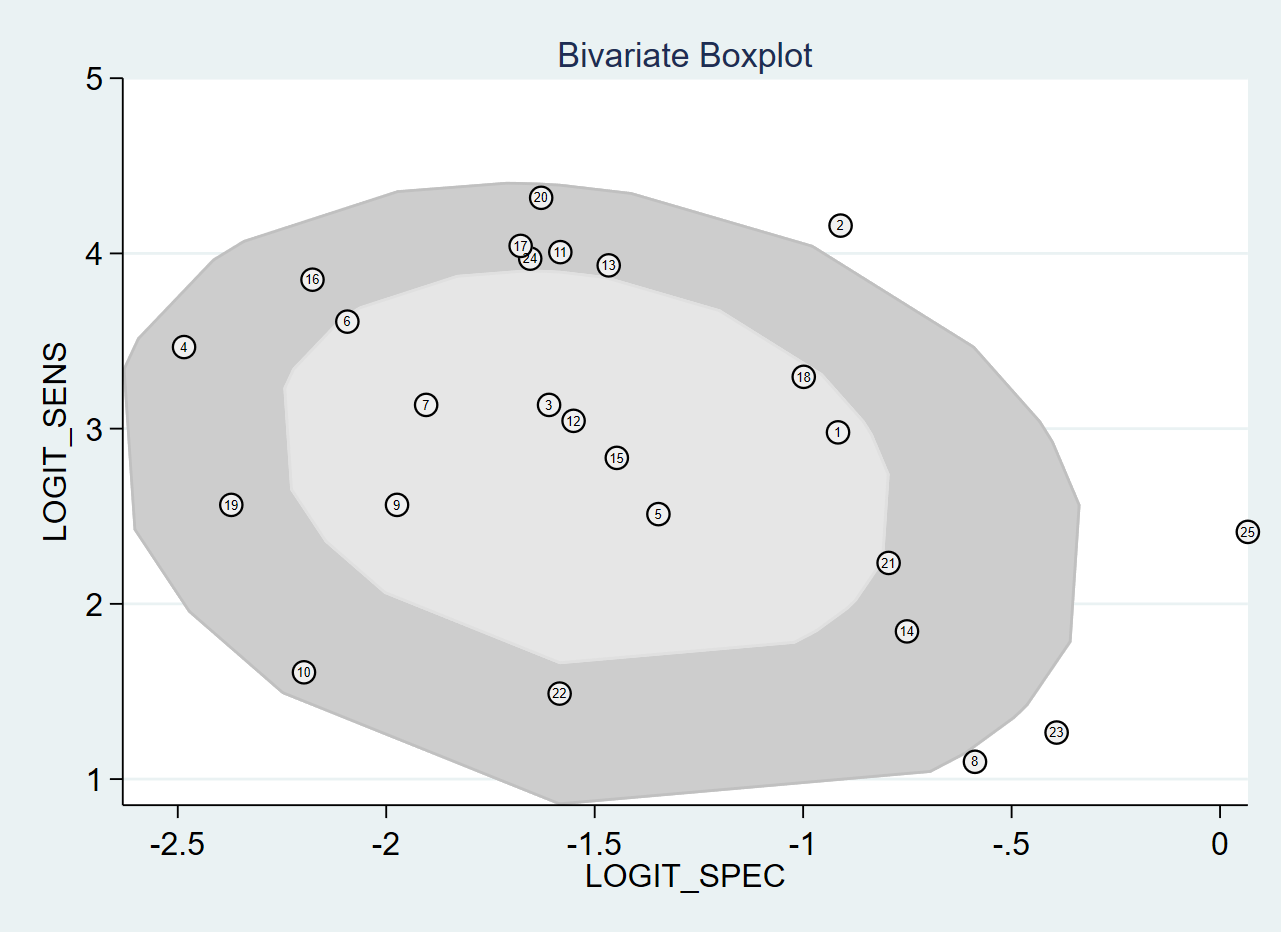


Supplementary Figure 12: Bivariate boxplot for Afirma GEC


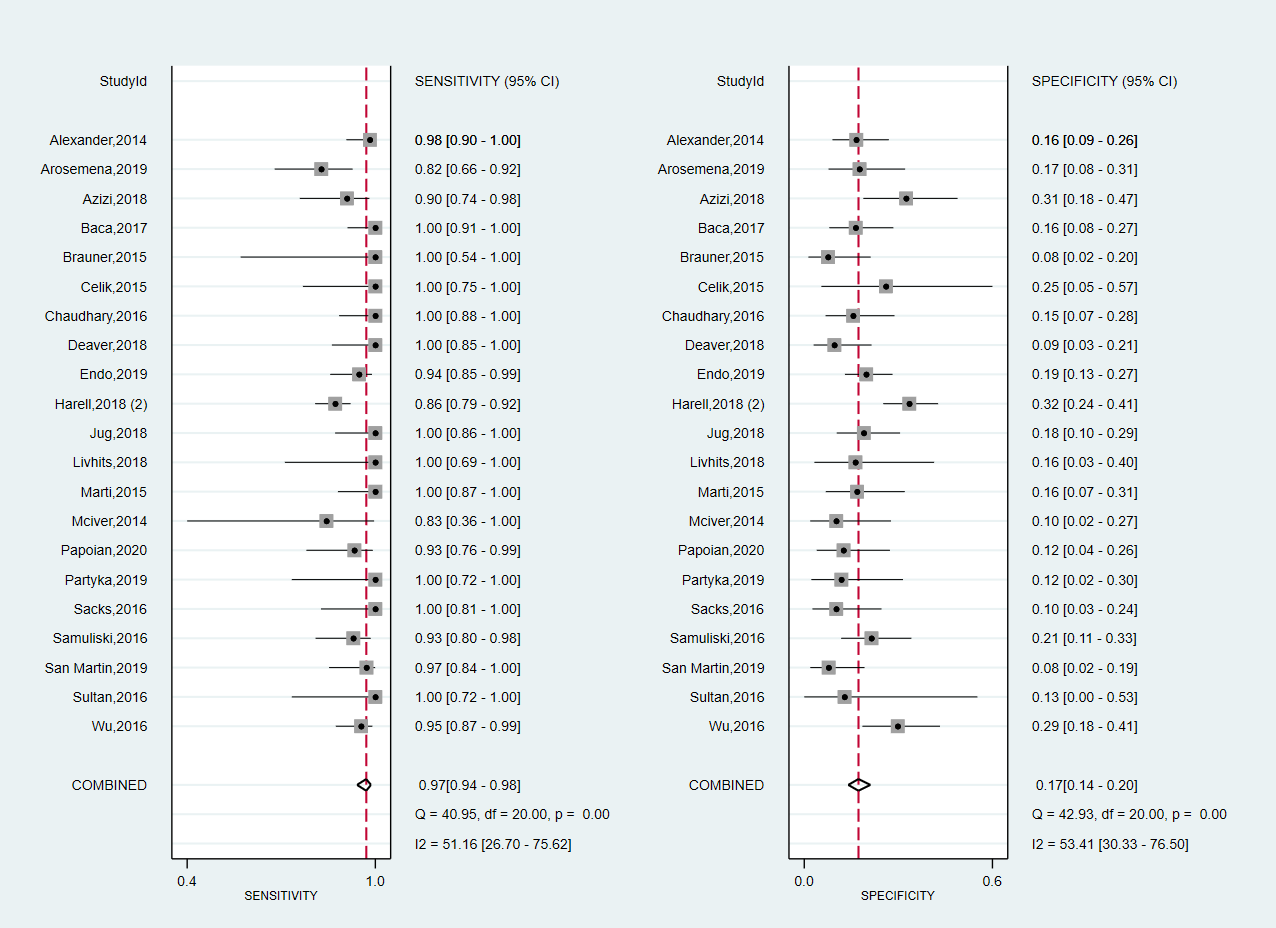


Supplementary Figure 13: Forest plot for Afirma GEC overall sensitivity and specificity excluding outliners


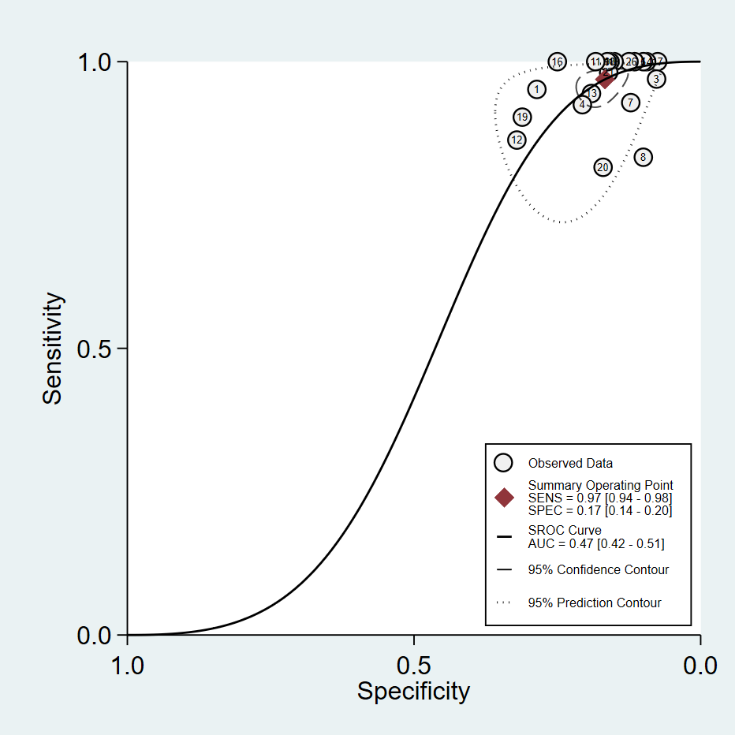


Supplementary Figure 14: SHROC curve for Afirma GEC overall sensitivity and specificity excluding outliners


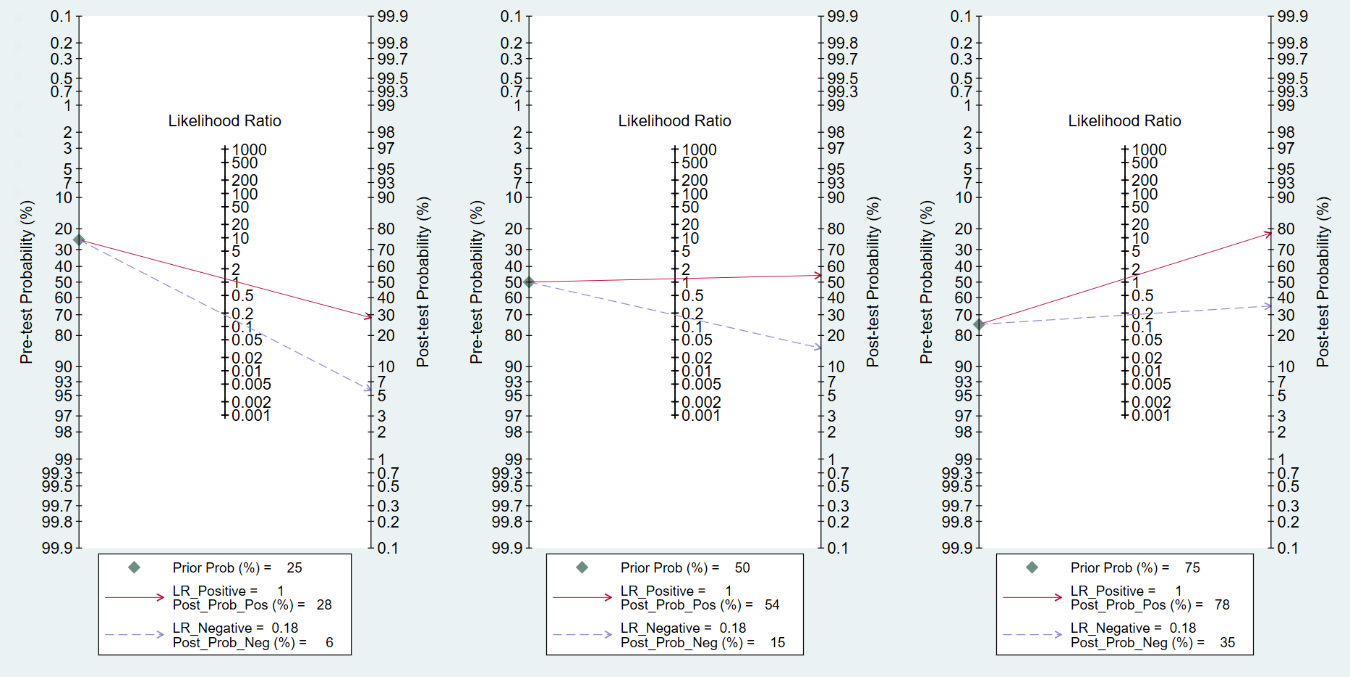


Supplementary Figure 15: Fagan’s nomogram for Afirma GEC


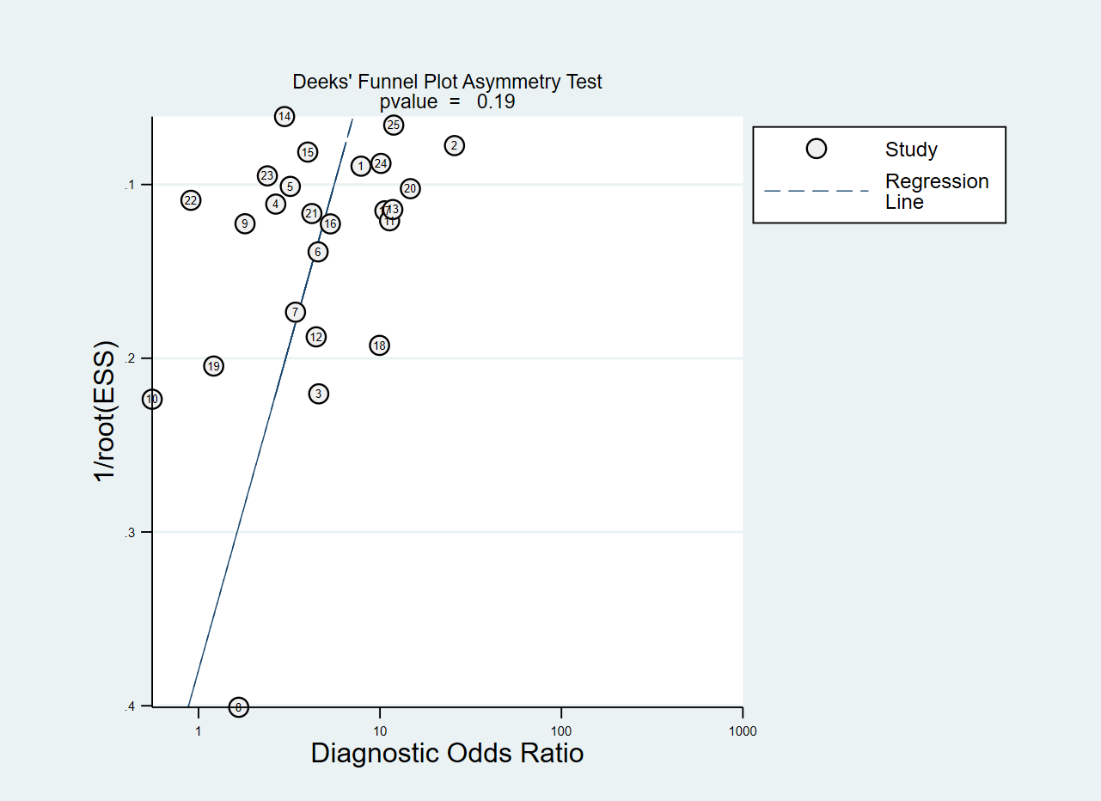


Supplementary Figure 16: Deek’s funnel plot asymmetry test for publication bias


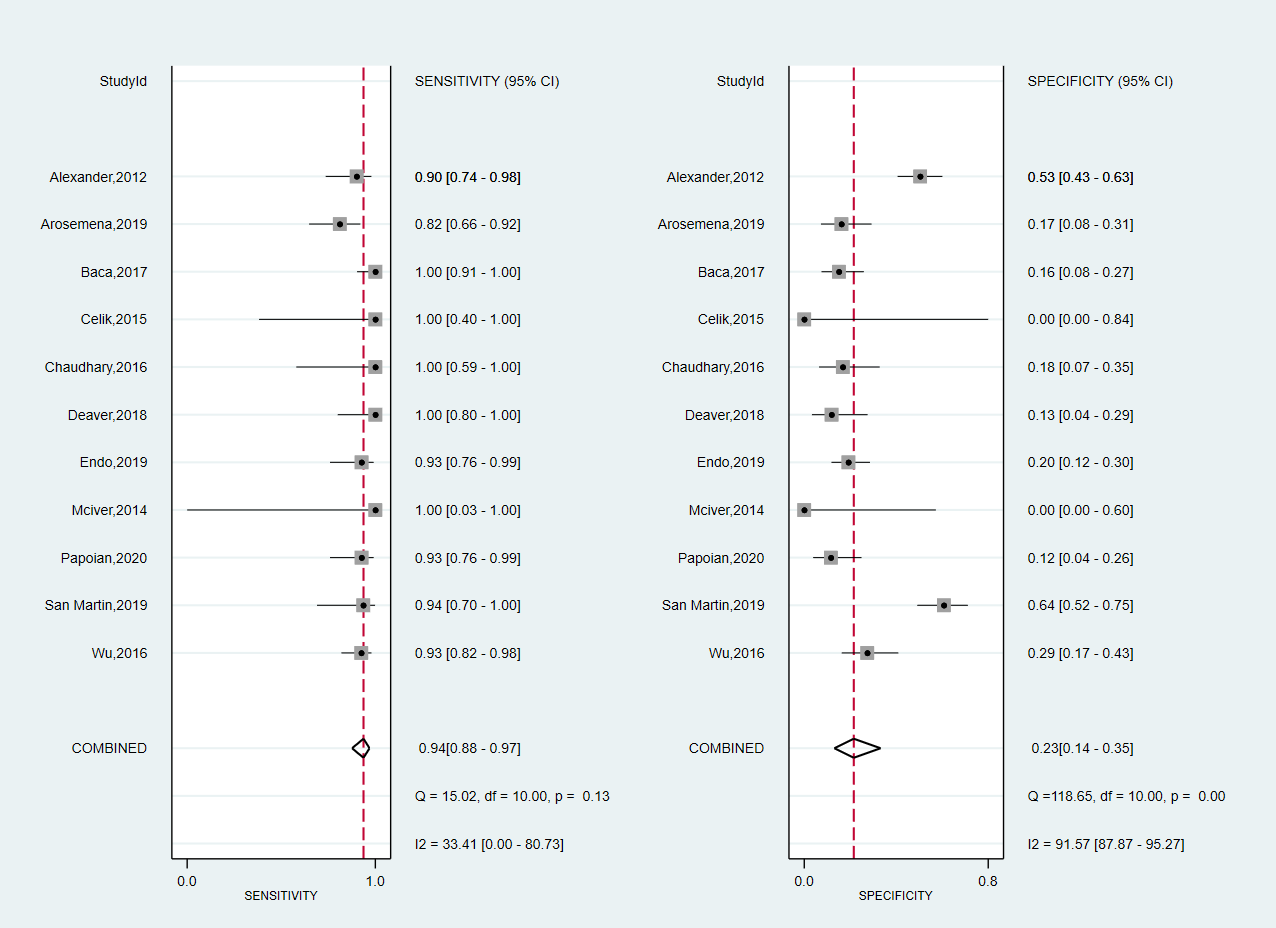


Supplementary Figure 17: Forest plot for Afirma GEC overall sensitivity and specificity in Bethesda III nodules


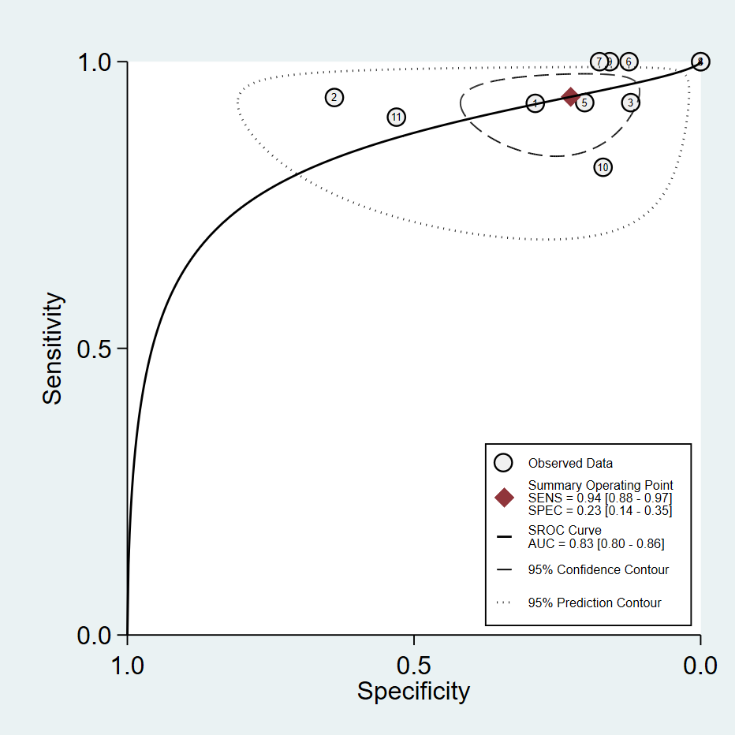


Supplementary Figure 18: SHROC curve for Afirma GEC overall sensitivity and specificity in Bethesda III nodules


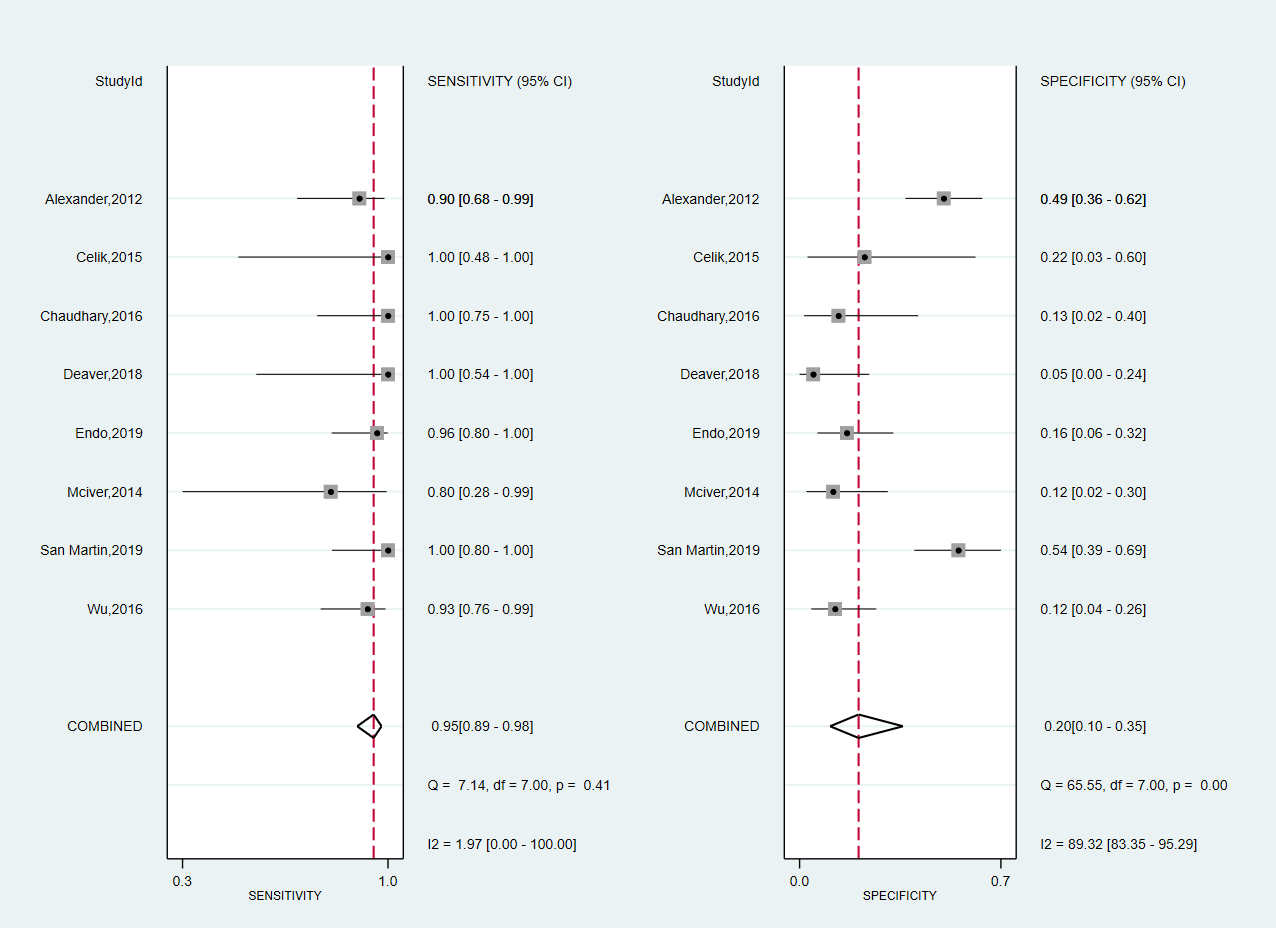


Supplementary Figure 19: Forest plot for Afirma GEC overall sensitivity and specificity in Bethesda IV nodules


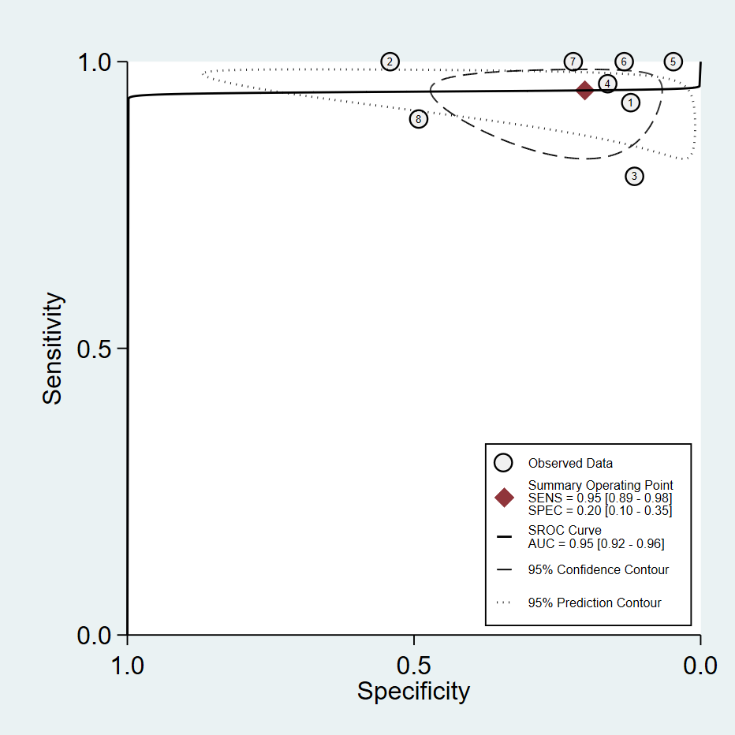


Supplementary Figure 20: SHROC curve for Afirma GEC overall sensitivity and specificity in Bethesda IV nodules


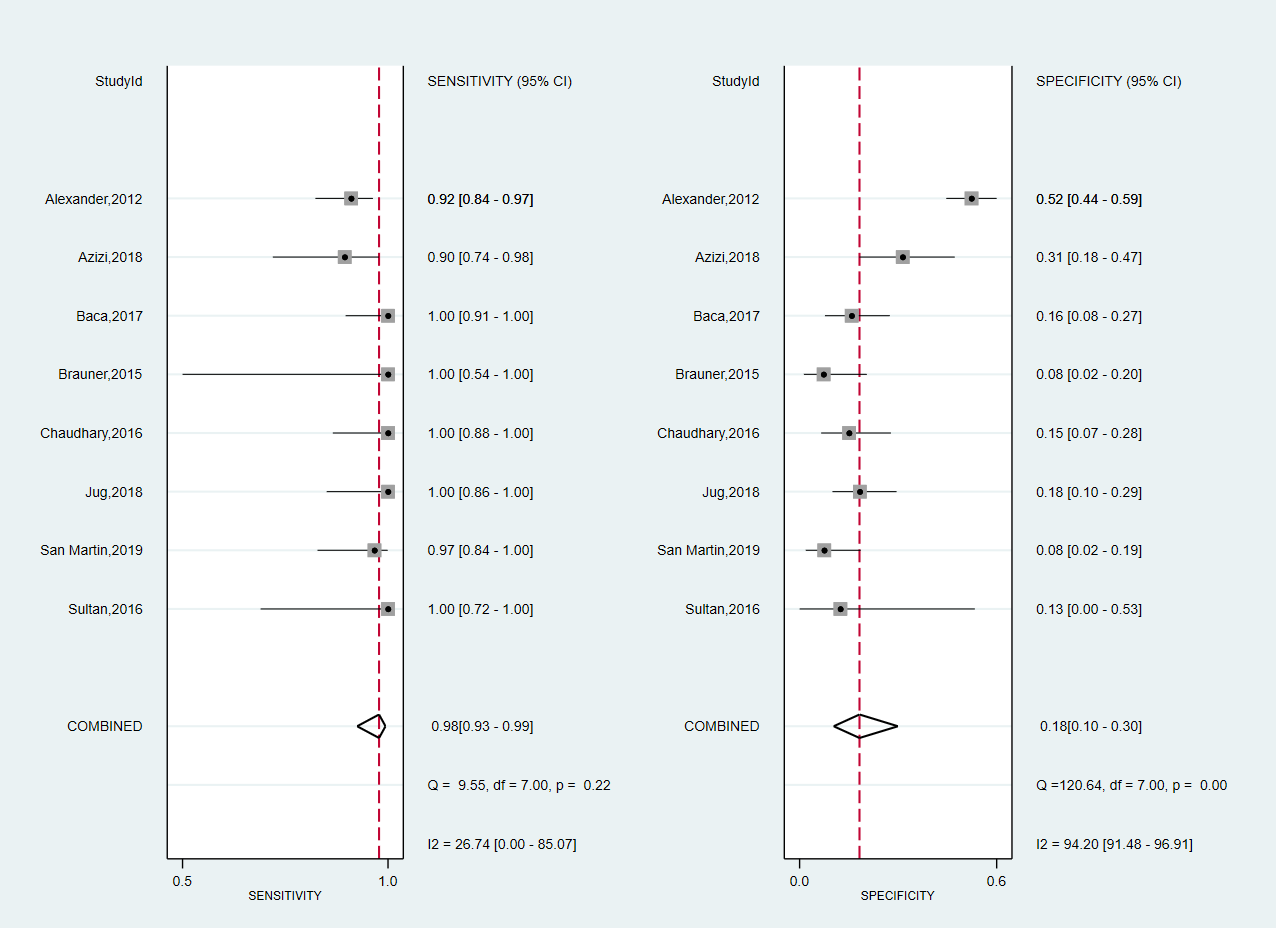


Supplementary Figure 21: Forest plot for Afirma GEC overall sensitivity and specificity including studies that confirmed ITNs by repeat FNA.


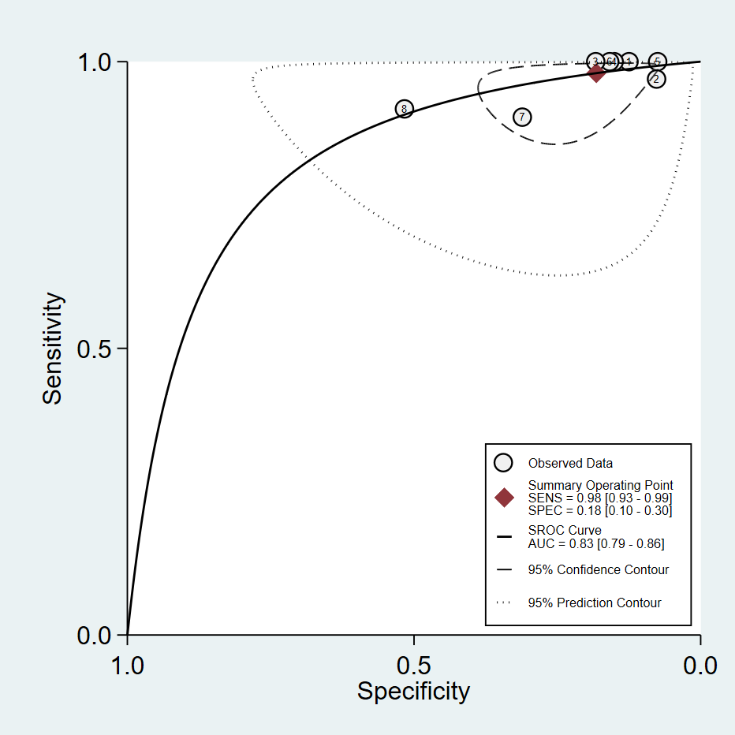


Supplementary Figure 22: SHROC curve for Afirma GEC overall sensitivity and specificity including studies that confirmed ITNs by repeat FNA.


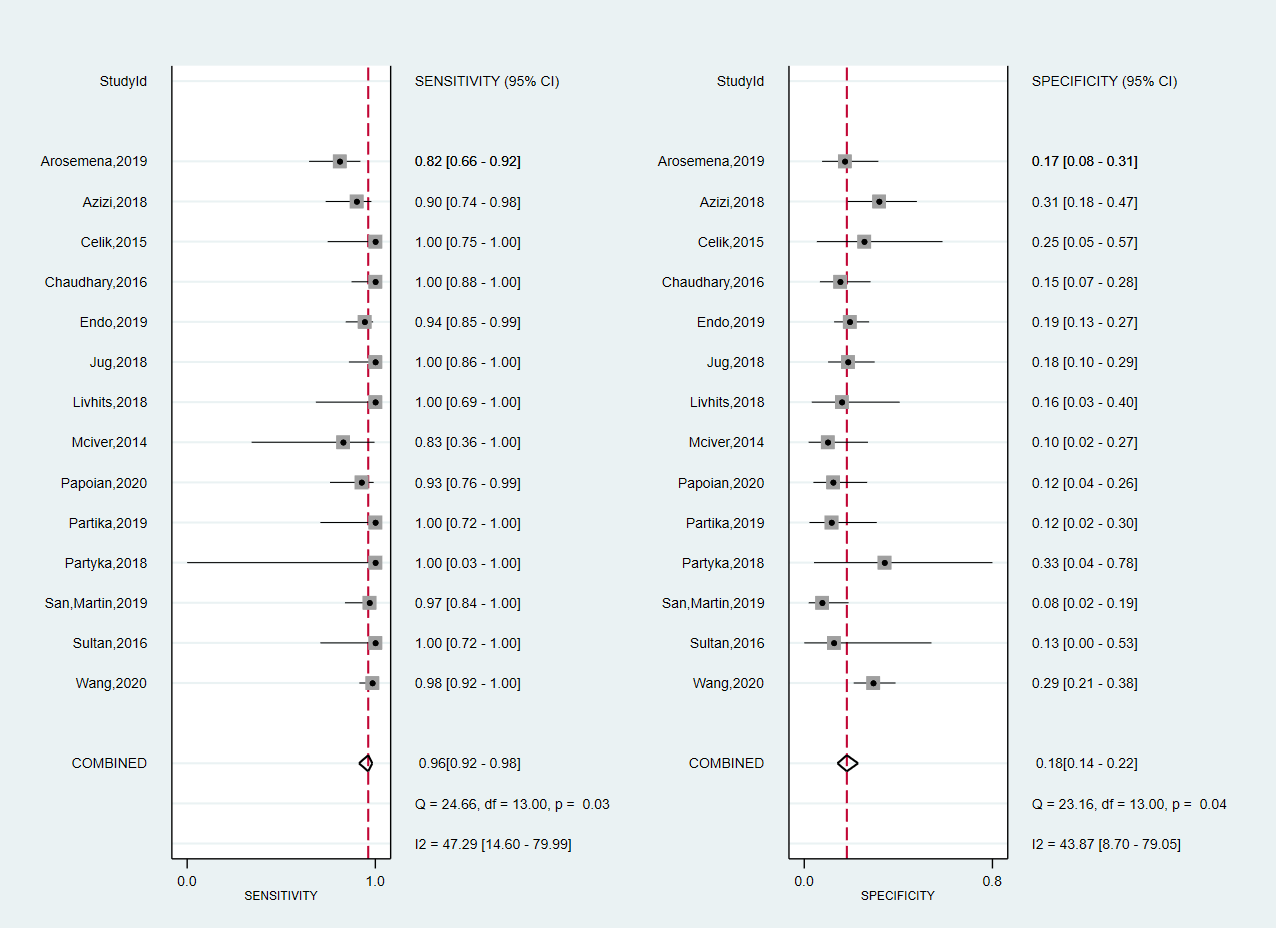


Supplementary Figure 23: Forest plot for Afirma GEC overall sensitivity and specificity including studies without declared conflicts of interests


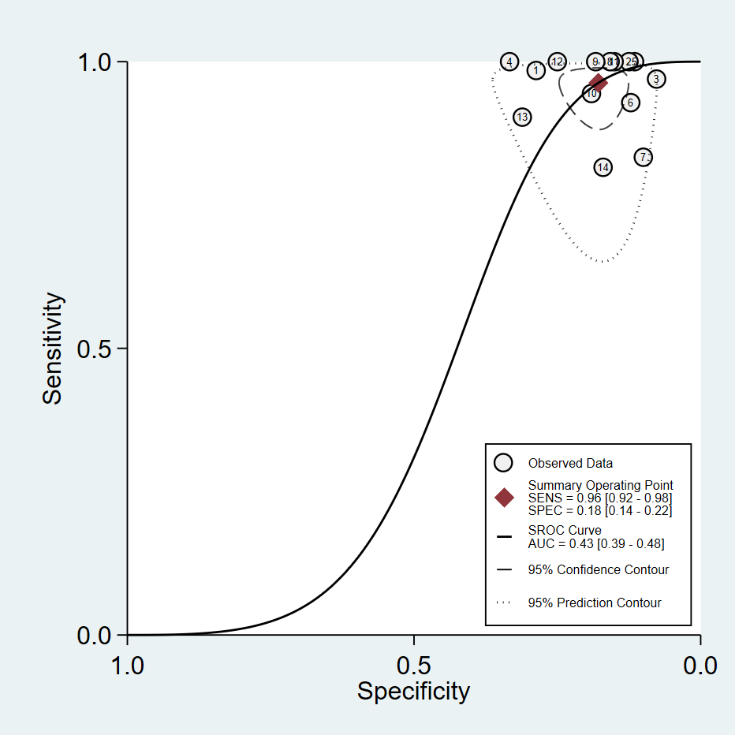


Supplementary Figure 24: SHROC curve for Afirma GEC overall sensitivity and specificity including studies without declared conflicts of interests


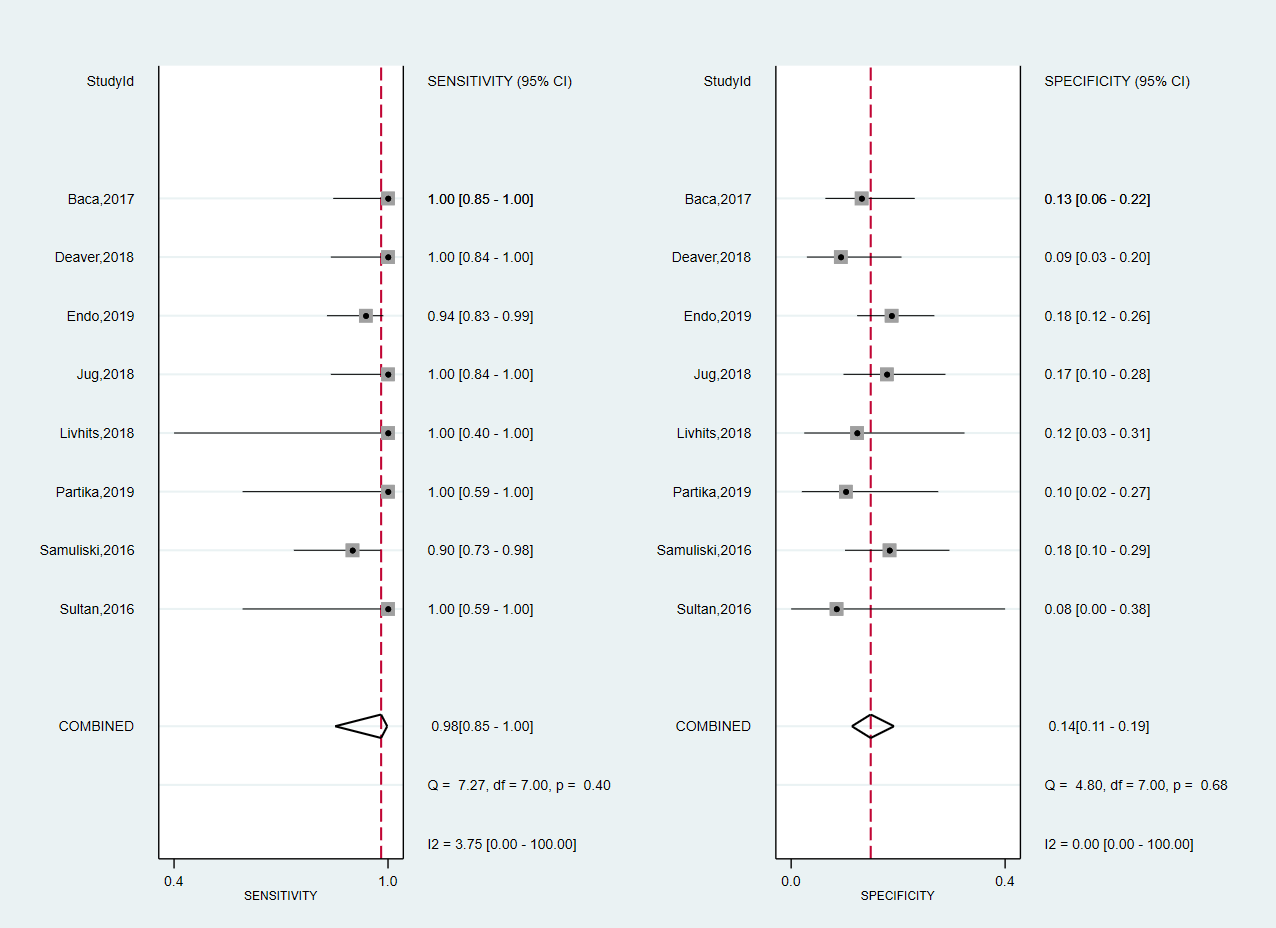


Supplementary Figure 25: Forest plot for Afirma GEC overall sensitivity and specificity when excluding NIFTPs from malignant tumors


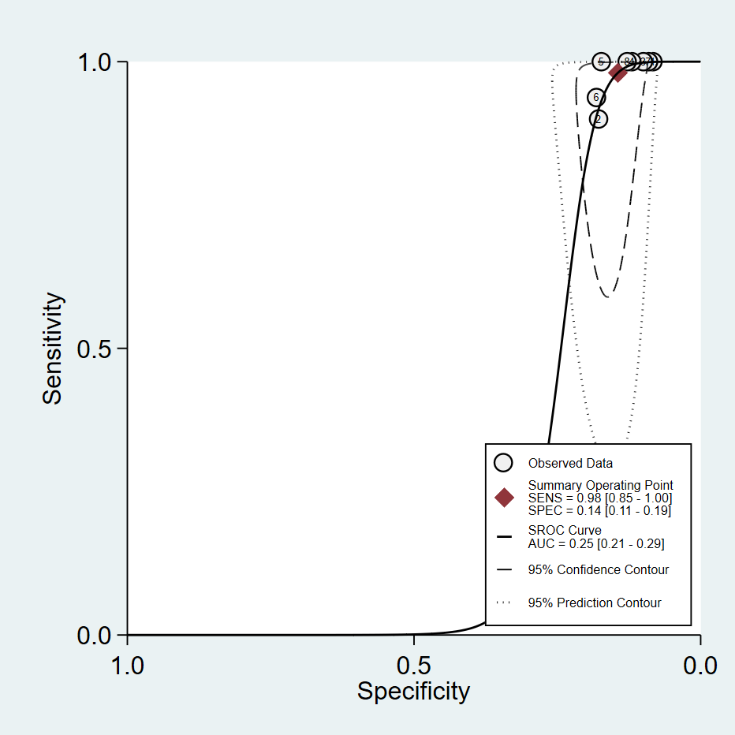


Supplementary Figure 26: SHROC curve for Afirma GEC overall sensitivity and specificity when excluding NIFTPs from malignant tumors


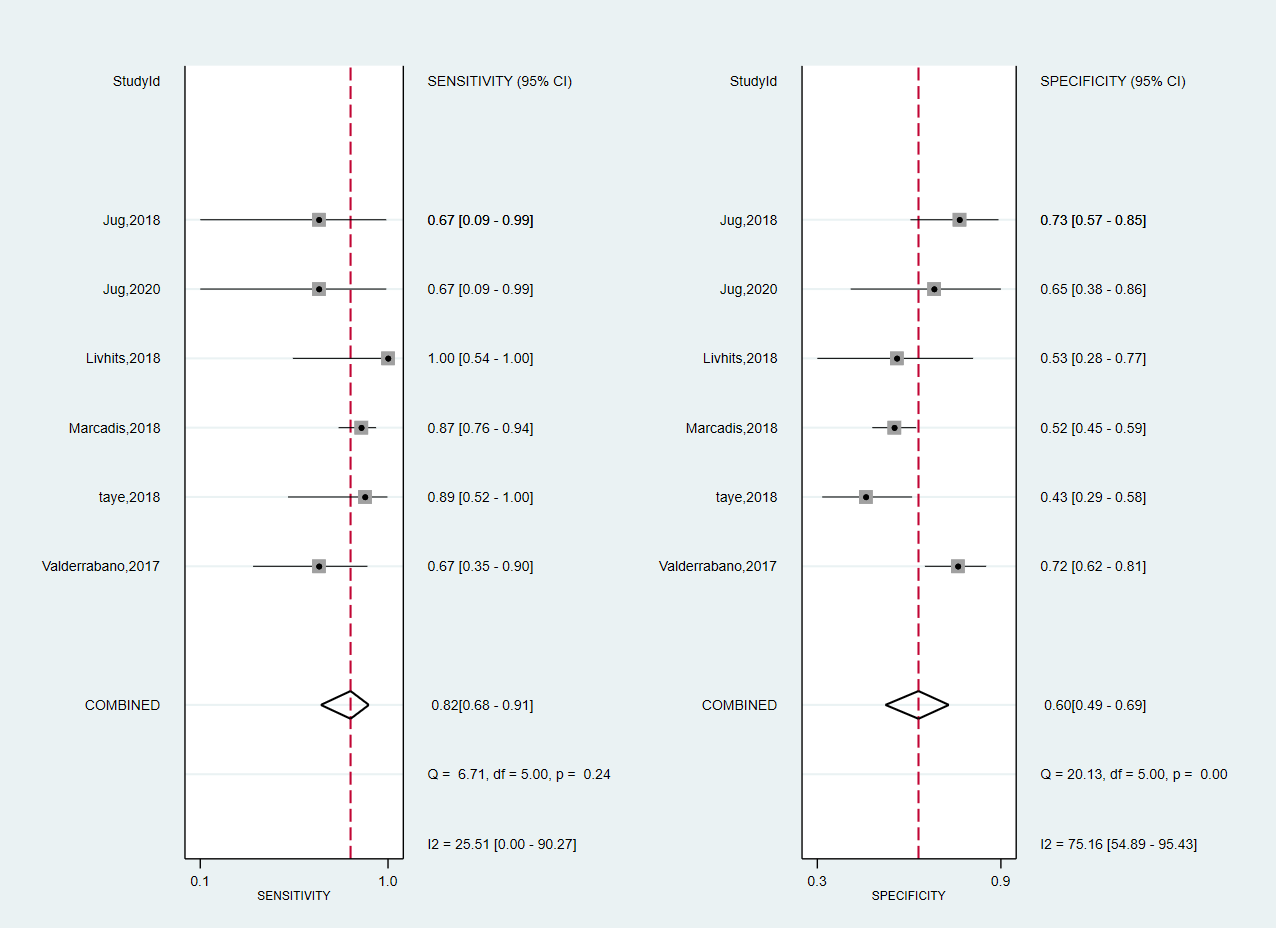


Supplementary Figure 27: Forest plot for Thyroseq 2 overall sensitivity and specificity when excluding NIFTPs from malignant tumors


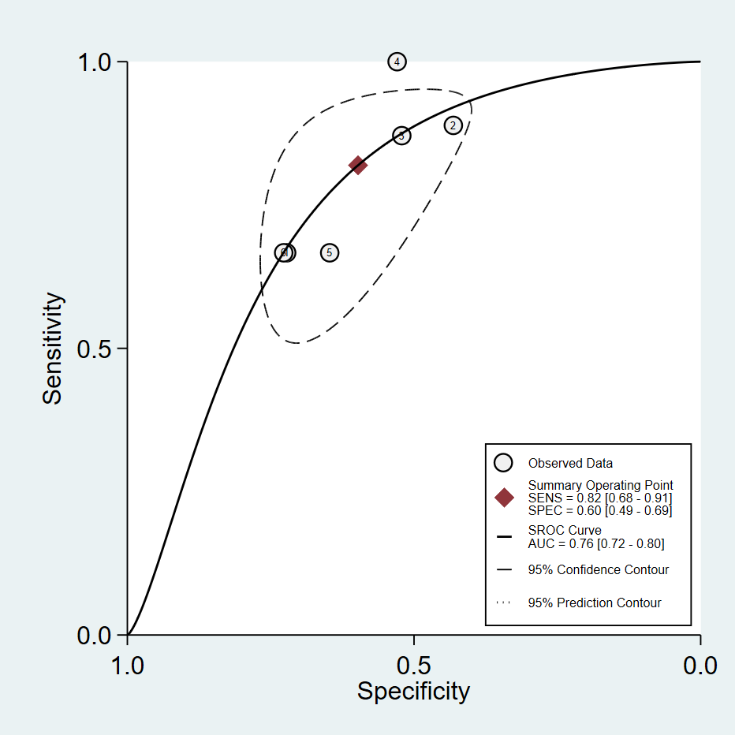


Supplementary Figure 28: SHROC curve for Thyroseq 2 overall sensitivity and specificity when excluding NIFTPs from malignant tumors

## Supplementary Tables

Supplementary Table 1: QUAPAS questionnaire.

| Domain | Description | Signaling question (yes, no, unclear) | Risk of bias (high, low, unclear) | Concerns about applicability (high, low, unclear) |
| --- | --- | --- | --- | --- |
| Participant recruitment | Describe the method for recruiting participants. Describe participants (previous testing, presentation, the intended use of index test and setting) | Was a consecutive or random sample of patients enrolled?  Was a case-control design avoided?  Did the study avoid inappropriate exclusions? | Could the selection of participants have introduced bias? | Are there concerns that the participants do Not match the review question? |
| Index test | Describe the index test and how it was conducted and interpreted: | Were the index test results interpreted without knowledge of the results of the reference standard?  Were all results of the index test valid? | Could the conduct or interpretation of the index test have introduced bias? | Are there concerns that the index test, its conduct, or its interpretation differ from the review question? |
| Reference standard | Describe the reference standard and how it was conducted and interpreted: | Is the reference standard likely to correctly classify the target condition?  Were the reference standard results interpreted without knowledge of the results of the index test? | Could the measurement of the target event have introduced bias? | Are there concerns that the target condition as defined by the reference standard does Not match the review question? |
| Flow and timing | Describe any patients who did Not receive the index test(s) and/or reference standard or who were excluded from the 2x2 table (refer to flow diagram): | Did all patients receive a reference standard?  Was available the reference standard for all the enrolled patients? | Could the patient flow have introduced bias? |  |

**Supplementary Table 2:** **QUADAS-2 signaling questions for bias.**

| **Question** | **Yes** | **Unclear** | **No** |
| --- | --- | --- | --- |
| **Participant recruitment** | | | |
| **Consecutive**  **or random sample enrolled?** | A consecutive or random sample of patients was enrolled in the  study. | It is unclear whether a consecutive or random sample of patients was enrolled in the study | There was No consecutive or random sample included in the study |
| **Case-control**  **design avoided?** | There was No case-control design | It is unclear if there was a case  control design | There was a case-control design |
| **Inappropriate exclusions**  **avoided?** | There are No patients inappropriate excluded | It is unclear if there was the avoidance of inappropriate exclusions | There is the inappropriate exclusion of patients (e.g. patients with inadequate or insufficient genetic material, patients with unkNown histology) |
| **Index test** | | | |
| **Index test results interpreted without knowledge results reference standard?** | The evaluators of the index test were unaware of the histological result. | It is unclear whether the evaluators of the index test were aware of the histological result. | The evaluators of the index test were aware of the histological result. |
| **Pre-specified threshold?** | This item will be omitted as each index test sets its pre-specified threshold. | | |
| **Were all results of the index test valid?** | At least 95% of the index test result were valid. | It is unclear the proportion of valid index test results. | Less than 95% of the index test result were valid. |
| **Reference standard** | | | |
| **Reference**  **standard likely to correctly classify the target**  **condition?** | There  is an adequate histopathological examination of thyroid tissue. | It is unclear how histopathological  the examination is performed. | The histopathological examination is Not  adequate. |
| **Reference standard results**  **interpreted**  **without the knowledge results in the index test?** | The outcome assessor of histopathological results was Not aware of molecular testing results | It is not clear if the outcome assessor of histopathological  results were aware of  molecular testing results | The outcome assessor of histopathological results was Not aware of molecular testing results |
| **Flow and timing** | | | |
| **The appropriate interval between index**  **test and reference standard?** | This item will be omitted as we considered the time interval to be irrelevant to the topic. | | |
| **All patients**  **received reference standard?** | All patients received surgery and histological result. | It is not clear if the whole sample did receive surgery or follow-up. | Some patients did not receive surgery but instead had a clinical follow-up. |
| **Patients received the same reference standard?** | This item will be omitted as studies with reference standards other than histological evaluation were excluded. | | |
| **All patients included in the analysis?** | More than 80% of the enrolled patients were included in the analysis | It is not clear if all patients were included in the analysis. | Less than 80% of the enrolled patients were included in the analysis (e.g. patients lost to follow-up) |

Supplementary Table 3: 'Risk of bias' and 'Applicability concerns' assessment according to QUADAS-2.

| **Molecular panel** | **Study** | **RISK OF BIAS** | | | | **APPLICABILITY CONCERNS** | | |
| --- | --- | --- | --- | --- | --- | --- | --- | --- |
|  |  | **PATIENT SELECTION** | **INDEX TEST** | **REFERENCE STANDARD** | **FLOW AND TIMING** | **PATIENT SELECTION** | **INDEX TEST** | **REFERENCE STANDARD** |
| Afirma GEC | Alexander 2012 | 🡫 | 🡫 | 🡩 | 🡫 | 🡩 | 🡩 | 🡩 |
|  | Alexander 2014 | 🡩 | 🡫 | ? | 🡫 | 🡩 | 🡩 | 🡩 |
|  | Al-qurayshi 2016 | ? | ? | ? | 🡫 | 🡩 | 🡩 | 🡩 |
|  | Arosemena 2019 | 🡫 | ? | ? | 🡫 | 🡩 | 🡩 | 🡩 |
|  | Azizi 2018 | 🡫 | ? | ? | 🡫 | 🡩 | 🡩 | 🡩 |
|  | Baca 2017 | 🡫 | ? | ? | 🡫 | 🡩 | 🡩 | 🡩 |
|  | Brauner 2015 | 🡫 | ? | ? | 🡫 | 🡩 | 🡩 | 🡩 |
|  | Celik 2015 | ? | 🡫 | ? | 🡫 | 🡩 | 🡩 | 🡩 |
|  | Chaudhary 2016 | ? | 🡫 | ? | 🡫 | 🡩 | 🡩 | 🡩 |
|  | Deaver 2018 | ? | ? | ? | 🡫 | 🡩 | ? | 🡩 |
|  | Endo 2019 | 🡫 | 🡫 | ? | 🡫 | ? | 🡩 | ? |
|  | Harell 2018 (2) | ? | ? | ? | 🡫 | 🡩 | 🡩 | ? |
|  | Jug 2018 | ? | ? | ? | 🡫 | ? | 🡩 | ? |
|  | Livhits 2018 | 🡩 | 🡫 | 🡩 | 🡫 | 🡩 | 🡩 | 🡩 |
|  | Marti 2015 | 🡫 | ? | ? | 🡫 | 🡩 | 🡩 | 🡩 |
|  | Mciver 2014 | 🡫 | ? | ? | 🡫 | 🡩 | 🡩 | 🡩 |
|  | Partyka 2018 | 🡫 | 🡩 | ? | 🡫 | 🡫 | 🡩 | 🡩 |
|  | Partika 2019 | ? | ? | ? | 🡫 | 🡩 | ? | 🡩 |
|  | Papoian 2020 | ? | 🡩 | ? | 🡫 | ? | 🡩 | ? |
|  | Samuliski 2016 | 🡩 | ? | ? | 🡫 | 🡩 | 🡩 | 🡩 |
|  | Sacks 2016 | 🡫 | ? | ? | 🡫 | 🡩 | 🡩 | 🡩 |
|  | San Martin 2019 | 🡩 | ? | ? | 🡫 | 🡩 | 🡩 | 🡩 |
|  | Sultan 2016 | ? | ? | ? | 🡫 | 🡩 | 🡩 | 🡩 |
|  | Wang 2020 | 🡫 | ? | ? | 🡫 | 🡩 | 🡩 | 🡩 |
|  | Wu 2016 | 🡫 | 🡫 | 🡩 | 🡫 | 🡩 | 🡩 | 🡩 |
| Afirma GSC | Harell 2018 (2) | ? | ? | ? | 🡫 | 🡩 | 🡩 | ? |
|  | Endo 2019 | 🡫 | ? | ? | 🡫 | 🡩 | 🡩 | 🡩 |
|  | Patel 2018 | 🡫 | 🡫 | 🡩 | 🡩 | 🡩 | 🡩 | 🡩 |
|  | San Martin 2019 | 🡩 | ? | ? | 🡫 | 🡩 | 🡩 | 🡩 |
| Thyroseq v3 | Chen 2019 | ? | 🡫 | ? | 🡫 | ? | 🡩 | 🡩 |
|  | Jug 2020 | ? | ? | ? | 🡫 | ? | 🡩 | 🡩 |
|  | Nikiforova 2018 | 🡫 | ? | ? | 🡩 | 🡩 | 🡩 | 🡩 |
|  | Steward 2018 | 🡫 | 🡫 | 🡩 | 🡩 | 🡩 | 🡩 | 🡩 |
| Thyroseq v2 | Jug 2018 | ? | ? | ? | 🡫 | 🡩 | 🡩 | ? |
|  | Livhits 2018 | 🡩 | ? | ? | 🡫 | 🡩 | 🡩 | 🡩 |
|  | Jug 2020 | ? | ? | ? | 🡫 | 🡩 | 🡩 | 🡩 |
|  | Marcadis 2018 | 🡫 | ? | ? | 🡩 | 🡩 | 🡩 | 🡩 |
|  | Nikiforov 2014 | 🡫 | ? | 🡩 | 🡩 | 🡩 | 🡩 | 🡩 |
|  | Nikiforov 2015 | 🡩 | ? | 🡫 | 🡫 | 🡩 | 🡩 | 🡩 |
|  | Shrestha 2016 | 🡫 | ? | ? | 🡩 | 🡩 | ? | ? |
|  | Taye 2018 | 🡫 | ? | 🡩 | 🡫 | 🡩 | 🡩 | 🡩 |
|  | ValderrabaNo 2017 | 🡩 | ? | 🡫 | 🡫 | 🡩 | 🡩 | 🡩 |
| Thyramir/ Thygenext (Interpace) | Lupo 2018 | 🡫 | 🡩 | 🡩 | 🡩 | 🡩 | 🡩 | 🡩 |
|  | Partyka 2018 | 🡫 | 🡩 | ? | 🡩 | 🡩 | 🡩 | 🡩 |
|  | Partyka 2019 | ? | ? | ? | 🡫 | 🡩 | 🡩 | 🡩 |
| RosettaGX Reveal | Litwick 2016 | 🡫 | 🡫 | 🡩 | 🡩 | 🡩 | 🡩 | 🡩 |
|  | Partyka 2018 | 🡫 | 🡩 | ? | 🡩 | 🡩 | 🡩 | 🡩 |
|  | Partyka 2019 | 🡫 | ? | ? | 🡩 | 🡩 | 🡩 | 🡩 |
| miRInform | Labourier 2015 | 🡫 | ? | 🡩 | 🡩 | 🡩 | 🡩 | 🡩 |
|  | ValderrabaNo 2016 | 🡩 | ? | 🡫 | 🡫 | 🡩 | 🡩 | 🡩 |

🡩Low-Risk 🡫High Risk ? Unclear Risk
